# Transcriptional repression by TGIF2 coordinates neurogenic priming and neural stem cell maintenance

**DOI:** 10.1126/sciadv.aea9974

**Published:** 2026-06-26

**Authors:** Yiling Li (李怡灵), Anthi C. Krontira, Franziska Vierl, Maria L. Richter, Weixu Wang (汪伟旭), Juliane Merl-Pham, Fabian J. Theis, Stefanie M. Hauck, Magdalena Götz

**Affiliations:** ^1^ Institute for Stem Cell Research, Helmholtz Center Munich, Biomedical Center, Planegg-Martinsried, Germany.; ^2^ Physiological Genomics, Biomedical Center, Medical Faculty, LMU Munich, Planegg-Martinsried, Germany.; ^3^ Graduate School of Systemic Neurosciences, LMU Munich, Planegg-Martinsried, Germany.; ^4^ Institute of Computational Biology, Computational Health Center, Helmholtz Munich, Munich, Germany.; ^5^ TUM School of Life Sciences Weihenstephan, Technical University of Munich, Freising, Germany.; ^6^ Research Unit Protein Science and Metabolomics and Proteomics Core, Helmholtz Center Munich, Neuherberg, Germany.; ^7^ School of Computation, Information and Technology, Technical University of Munich, Munich, Germany.; ^8^ Excellence Cluster of Systems Neurology (SyNergy), Biomedical Center, LMU Munich, Planegg-Martinsried, Germany.

## Abstract

Stem cells balance self-renewal with differentiation. Priming, i.e., low-level expression of differentiation-related genes, enables stem cells to remain undifferentiated and progeny to fast proceed in up-regulation of differentiation genes. Here, we investigated transcriptional priming during neurogenesis to search for key regulators. Assay for transposase-accessible chromatin using sequencing (ATAC-seq) and RNA sequencing of neural stem cells (NSCs) and neurons across forebrain regions identified hundreds of neuronal differentiation genes open while lowly expressed in NSCs. Pantelencephalic candidate transcription factors regulating neurogenic primed genes highlighted the transcriptional repressor transforming growth factor–β–induced homeobox factor 2 (TGIF2). We show that TGIF2 binds and represses neuronal differentiation genes in NSCs by interacting with SIN3 transcription regulator family member A/histone deacetylase corepressor complexes. Gain- and loss-of-function experiments in vitro and in vivo show that TGIF2 maintains NSC identity, slows neuronal differentiation, and regulates more than half of the primed neuronal differentiation genes in NSCs. Together, our database determines primed gene expression across regions and stages and identifies TGIF2 as a key regulator of neurogenic NSC fate.

## Teaser

TGIF2 acts as a key repressor that balances neural stem cell maintenance and differentiation.

## INTRODUCTION

Stem cells choose among different fates for their progenies while balancing differentiation versus self-renewal. To achieve this, stem cells can use lineage priming, by which they begin preparing for specific fate decisions before full lineage commitment. The regulation of lineage priming helps stem cells rapidly respond to differentiation signals while preserving plasticity. Priming can occur at the epigenetic, transcriptional, and translational levels (*1*–*3*). At the epigenetic level, lineage-specific regulatory elements acquire certain epigenetic marks, such as chromatin marks mediated by disrupter of telomeric silencing 1-like (DOT1L) or other histone methyltransferases often referred to as “poised transcription” (*4*–*7*). This poised and repressed state can be mediated by polycomb repression and RNA polymerase II stalling (*8*). At the transcriptional level, regulatory regions of genes are accessible, while their gene expression remains low, achieving a primed but inactive state. For example, in hematopoietic stem cells, early chromatin opening at lymphoid gene loci biases lineage output toward lymphoid fates without full gene activation (*3*). At the translational level, specific mRNAs can be transcribed but remain translationally repressed until extrinsic cues initiate their rapid translation (*1*). In neural stem cells (NSCs), all three layers of priming—epigenetic (*9*), transcriptional (*2*), and translational (*1*)—contribute to progenitor heterogeneity.

However, still very little is known about the transcription factors (TFs) governing these priming states. Canonical pathways such as NOTCH and repressor element-1 silencing transcription factor (REST) have been implicated in cell fate transitions of NSCs (*10*–*12*), but these findings do not fully explain how transcriptional programs are selectively repressed yet primed. In particular, how transcriptional repressors contribute to lineage priming without triggering full differentiation remains an open question (*1*).

While much research has focused on transcriptional activators driving neurogenesis, transcriptional repressors that preserve progenitor identity and restrain premature differentiation are comparatively understudied. Notably, several repressors—such as methyl-CpG-binding protein 2 (Mecp2), zinc finger E-box-binding homeobox 2 (Zeb2), and chromodomain helicase DNA binding protein 4 (Chd4) (*13*–*15*)—are implicated in severe neurodevelopmental disorders, highlighting their functional importance in brain development. However, to which extent these factors are involved in regulating transcriptional priming is not known. This raises a fundamental question: Are there central transcriptional repressors coordinating neurogenic priming and NSC maintenance, thereby ensuring proper progression of neurogenesis and the correct cortical cytoarchitecture?

## RESULTS

### Identification of neurogenic primed genes across forebrain regions

**Fig. 1.**
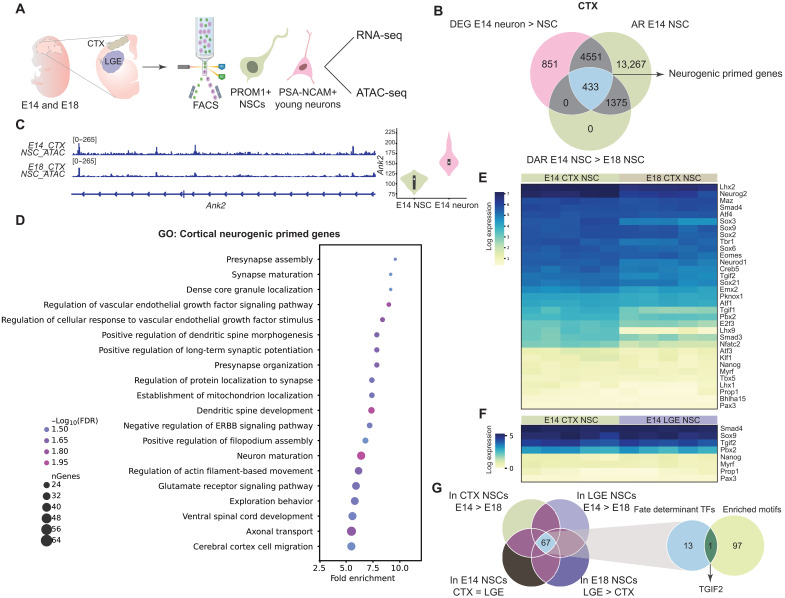
Screening of neurogenic primed genes and their regulators. (**A**) Experimental scheme of RNA-seq and ATAC-seq. PROM1+, PROMININ1. (**B**) Venn diagram showing data mining logic of neurogenic primed genes. (**C**) Example of ATAC-seq profiles in E14 and E18 cortical NSCs of a neurogenic primed gene, *Ank2*, together with its RNA expression by violin plot in E14 cortical NSCs and neurons. (**D**) Gene Ontology (GO) terms associated with biological processes for cortical neurogenic primed (CNP) genes, showing top 20 terms. ERBB, erythroblastic oncogene B. (**E**) Expression heatmap of candidate regulators of neurogenic primed genes in E14 and E18 cortical NSCs. (**F**) Expression heatmap of candidate regulators of neurogenic primed genes filtered for nonregion specific TFs in E14 NSCs between cortex (CTX) and LGE. (**G**) Venn diagram of neurogenic NSC regulators identified from the transcriptome analysis and the differentially enriched motifs.

To identify primed genes and their potential regulators in neurogenesis, it is important to analyze NSCs at the peak of neurogenesis, embryonic day 14 (E14), in the murine cerebral cortex and compare their expression profiles to the onset of gliogenesis, E18, when lineage priming changes toward glial lineages. Therefore, NSCs were isolated by their cell surface protein Prominin1+ from the cerebral cortex and lateral ganglionic eminence (LGE) at these stages, following previously established protocols (Fig. 1A) (*16*). Notably, neurogenesis of olfactory bulb interneurons continues into adulthood in the LGE, suggesting that these NSCs may maintain neurogenic lineage priming for these neurons at E18. To characterize neuronal gene expression, we also isolated neurons by polysialic acid (PSA)–neural cell adhesion molecule (NCAM)+ at E14 and performed deep bulk RNA sequencing (RNA-seq) and assay for transposase-accessible chromatin using sequencing (ATAC-seq) (Fig. 1A). Principal components analysis (PCA) revealed that brain region and developmental stage accounted for more than 70% of the variance in both RNA-seq and ATAC-seq datasets (fig. S1, A and B), while technical parameters had minimal impact (fig. S1, A to D).

To define neurogenic primed genes, we selected genes with significantly higher expression in E14 cortical neurons compared to NSCs [5835 genes at false discovery rate (FDR): 5%]. Of these, 85.4% (4984 genes) already exhibited accessible chromatin, i.e., were associated to a peak signal in ATAC-seq, in E14 NSCs—consistent with a transcriptionally primed but inactive state (Fig. 1B). Notably, 60% of these were inside genic regions, while the remainder were more distal to the associated gene. To further ensure their identity as primed genes, we intersected this set with genes, for which the associated chromatin accessibility decreased in E18 cortical NSCs during gliogenic transition (Fig. 1B). This approach identified 433 genes, hereafter referred to as cortical neurogenic primed (CNP) genes (Fig. 1, B and C, and table S1). Gene Ontology (GO) analysis revealed enrichment for neuronal differentiation and maturation processes, including “axonogenesis” and “synapse assembly” (Fig. 1D). These findings indicate that cortical NSCs harbor a broad neurogenic program marked by accessible chromatin well before full transcriptional activation. CNP genes also showed higher expression in E14 cortical neurons compared to nonprimed genes (fig. S1, E and F), suggesting that these genes can be activated faster upon differentiation cues.

To uncover regulators of CNP genes, we performed motif search analysis on the genomic regions that became reduced in accessibility in E18 compared to E14 cortical NSCs (i.e., during gliogenic transition) and were associated to the CNP genes. In addition, we filtered for TFs with higher mean expression in E14 cortical NSCs than in E18 cortical NSCs (Fig. 1E). This strategy yielded 33 candidate TFs that may either activate or repress neurogenic primed genes (table S1). As we noted many region-specific TFs (e.g., Neurogenin 2), we further filtered out the TFs that were differentially expressed between NSCs in cortex and LGE at E14. This yielded eight TFs (Fig. 1F), which can be considered as more panneurogenic, or at least pantelencephalic, neurogenic priming factors. Of note, amongst these eight, Smad4, Sox9, Tgif2, and Pbx2 are on average around 12 times more highly expressed than the other four.

### Identification of TFs in regulating neurogenic NSC fate

To identify TFs that may more broadly regulate the neurogenic state of NSCs, we focused on neurogenic NSCs at E14 in cortex and LGE and compared them to NSCs starting to convert to gliogenesis (E18). Comparing cortex NSCs between E14 and E18, we identified extensive transcriptional and chromatin changes. We found 7455 differentially expressed genes (DEGs; 1% FDR; 33.5% of 22,215 detected genes; table S2 and fig. S2A). In the LGE, 8517 DEGs were found (1% FDR; table S2 and fig. S2B), constituting 38.33% of detected genes. Comparing the two regions at E18, when the cortex has transitioned to gliogenesis, while the LGE continues neurogenesis of a subset of neurons, we found 7330 DEGs (1% FDR; 32.9% of 22,215 detected genes; table S2 and fig. S2C). Chromatin remodeling was also very prominent in these comparisons, with 7654 differentially accessible regions (DARs) in cortex between the two time points (table S3 and fig. S2D). These were fewer (4710) in the LGE, where gliogenesis also starts, but neurogenesis of olfactory bulb (OB) interneurons continues (table S4 and fig. S2E). Comparing DARs at E18 between the two regions revealed 9576 DARs (table S5 and fig. S2F), underscoring the massive difference between a region ending neurogenesis and one continuing it, at least in part. This illustrates the power of this direct comparison that has not been performed so far.

To identify TFs involved in regulating the neurogenic state of NSCs, we focused on TFs more highly expressed in E14 than in E18 NSCs from both cortex and LGE and more highly expressed in E18 LGE NSCs than E18 cortical NSCs, given that LGE NSCs continue neurogenesis longer. To avoid factors involved in regional specification of cortex versus LGE, we excluded those differentially expressed between the two regions at E14 (1% FDR cutoff). Following this rationale, we found 67 genes (table S6), 14 of which are TFs and/or chromatin remodelers (Fig. 1G and table S6). To further prioritize functional regulators, we assessed motif accessibility using ATAC-seq. We searched for motifs that were more accessible in E14 cortex NSCs compared to E18 cortex and in E18 LGE compared to E18 cortex, excluding DARs between cortex and LGE at E14. This genomic analysis mirrors the transcriptomic criteria but excluding the E14 versus E18 LGE comparison, as the LGE continues neurogenesis and many neurogenic regions might not close at E18. Thereby, we identified 98 enriched TF motifs in neurogenic NSCs (table S7). Overlapping these with the 14 TFs from the expression analysis revealed a single shared TF, whose expression and motif accessibility were enriched in neurogenic NSCs: transforming growth factor–β (TGFβ)–induced homeobox factor 2 (TGIF2) (Fig. 1G).

As TGIF2 also emerged as a candidate for regulating neurogenic primed genes (Fig. 1F and table S1) and was reported as a repressor in cancer studies (*17*, *18*), we set out to explore its function in neurogenesis. In the nervous system, TGIF2 has only been examined in adult neurons in the context of autism spectrum disorder, where its overexpression (OE) alleviated behavioral phenotypes in mouse models (*19*). However, its function during brain development has not been explored in contrast to its family member TGIF1 that has been involved in holoprosencephaly (*20*). Consistent with our RNA-seq data, in situ hybridization shows that TGIF2 expression is strongly enriched in the ventricular zone, the germinal niche where NSCs reside (fig. S3A), and gradually down-regulated in development (fig. S3B). These findings suggest that TGIF2 may serve a previously unrecognized function in neurogenesis, particularly by regulating neurogenic priming programs and the maintenance of NSCs.

### TGIF2 promotes NSC maintenance in vitro

**Fig. 2.**
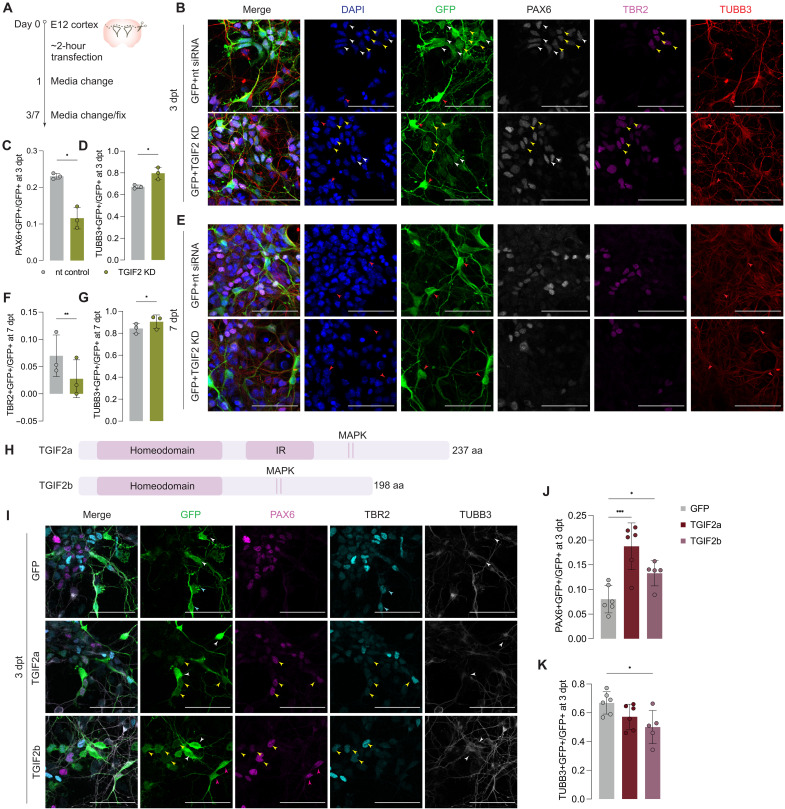
TGIF2 KD promotes differentiation, while TGIF2 OE promotes NSC state in vitro. (**A**) Schematic drawing showing the procedure of E12 cortical culture transfection assay. (**B** and **E**) Representative images showing transfected E12 cortical cultures in LOF experiment at 3 or 7 days posttransfection (dpt), respectively. White arrowheads for paired box 6+T-Box brain protein 2– (PAX6+TBR2-)GFP+/GFP+ cells, yellow arrowheads for PAX6+TBR2+GFP+/GFP+ cells, and red arrowheads for TUBULIN bIII (TUBB3)+GFP+/GFP+ cells. Scale bars, 50 μm. (**C**, **D**, **F**, and **G**) Quantifications of PAX6+GFP+/GFP+ and TUBB3+GFP+/GFP+ in LOF experiment at 3 or 7 days posttransfection, respectively; mean ± SD. *N* = 3 pools of embryos. Two-tailed paired *t* test. *P < 0.05 and **P < 0.01. (**H**) Schematic drawing of TGIF2 isoforms. IR, intron retention; aa, amino acids. (**I**) Representative images showing transfected E12 cortical cultures in GOF experiment at 3 or 7 days posttransfection, respectively. Magenta arrowheads for PAX6+TBR2–GFP+/GFP+ cells, cyan arrowheads for TBR2+PAX6–GFP+/GFP+ cells, yellow arrowheads for PAX6+TBR2+GFP+/GFP+ cells, and white arrowheads for TUBB3+GFP+/GFP+ cells. Scale bars, 50 μm. (**J** and **K**) Quantifications of PAX6+GFP+/GFP+ and TUBB3+GFP+/GFP+ at 3 days posttransfection; mean ± SD. *N* = 5 to 6 pools of embryos. Ordinary one-way analysis of variance (ANOVA) with Holm-Šídák’s multiple-comparison test. *P < 0.05 and ***P < 0.001.

To investigate the function of TGIF2 in neurogenesis, we performed loss- and gain-of-function (LOF/GOF) experiments in vitro, using dissociated cultures from E12 mouse cerebral cortex, a developmental stage enriched in NSCs (Fig. 2A). TGIF2 has two isoforms, TGIF2a and 2b, with the longer, intron-retaining isoform TGIF2a (Fig. 2H) being both the canonical variant and also the more abundantly expressed form during cortical development (fig. S3C) (*21*). For LOF, a pool of small interfering RNAs (siRNAs) targeting both isoforms of TGIF2 (Fig. 2B), will now be referred to as TGIF2 knockdown (KD), were cotransfected with a plasmid expressing green fluorescent protein (GFP) to visualize transfected cells. As the siRNAs are very small, they transfect almost all cells, and therefore, every GFP+ cell highly likely contains siRNA. The KD condition was compared to a pool of nontargeting siRNAs as controls (Fig. 2, B to G, and fig. S3, D to F). To validate siRNA KD efficiency, we cotransfected them with bicistronic expression plasmids encoding GFP and FLAG-tagged TGIF2a or TGIF2b in Neuro2a (N2A) cells. TGIF2 protein levels were subsequently assessed by Western blot (fig. S3D). Similar validations were performed in E12 cortical cultures overexpressing TGIF2a using antibodies against TGIF2 and the FLAG tag (fig. S3, E and F). These data showed significant reduction of both TGIF2a and TGIF2b to about half (fig. S3, D to F).

To explore whether TGIF2 KD would have any functional consequences on cell type identity, we fixed E12 cortex dissociated cell cultures 3 days posttransfection and immunostained for paired box 6 (PAX6), labeling NSCs, T-Box brain protein 2 (TBR2), specific for neuronal progenitors (NPCs), and TUBULIN bIII (TUBB3) to detect differentiating neurons (Fig. 2B). TGIF2 KD significantly reduced the proportion of PAX6+ NSCs (Fig. 2C) and increased TUBB3+ neurons (Fig. 2D), indicating that loss of TGIF2 promotes premature neuronal differentiation. When cell cultures were fixed at 7 days posttransfection and stained for the same marker proteins (Fig. 2E), we observed very few PAX6+ cells (fig. S3, G and H), as cells had differentiated further, possibly also due to dilution of the siRNAs in further divisions. However, TBR2+ progenitor cells were now significantly reduced (Fig. 2F), and TUBB3+ neurons were still significantly increased upon TGIF2 KD (Fig. 2G).

For GOF, we transfected E12 cortex cells with constructs encoding the two splice variants of TGIF2 (Fig. 2H). Constructs encoded triple-Flag–tagged TGIF2a or TGIF2b coexpressing GFP under the CAG promoter. A monocistronic GFP vector served as the negative control. OE of both forms of TGIF2 significantly increased the proportion of PAX6+ NSCs at 3 days posttransfection, with TGIF2a exerting a stronger effect than TGIF2b (Fig. 2, I and J). In parallel, the proportion of TUBB3+ neurons was significantly reduced in the TGIF2b condition (Fig. 2, I and K). Thus, TGIF2 OE maintains more NSCs and reduces neuronal differentiation.

To assess longer-term effects of TGIF2, we analyzed cultures at 7 days posttransfection. While PAX6+ cells are very few at this stage and plasmids are diluted when cells continue to proliferate, we now observed a significant increase in the proportion of TBR2+ NPCs in TGIF2a-OE cultures, with a similar but milder trend in TGIF2b-expressing cells (fig. S4, A to C). No significant difference in the proportion of neurons was observed at this later stage (fig. S4D). These results demonstrate that TGIF2, particularly TGIF2a, delays differentiation and sustains initially NSC states, followed by an increase in neuronal progenitor cells. Conversely, TGIF2 KD accelerates neuronal differentiation, reducing first PAX6+ NSCs and at later stages TBR2+ NPCs.

### TGIF2 function is dependent on its repressor domain and phosphorylation

**Fig. 3.**
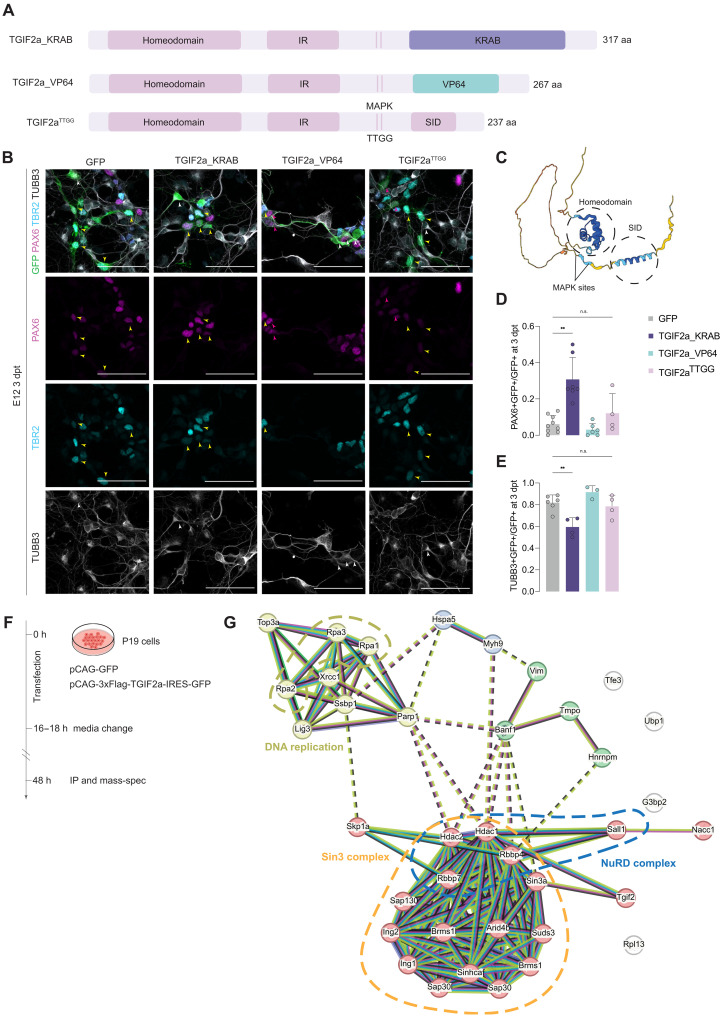
TGIF2 acts as a repressor and interacts with SIN3A corepressor complex. (**A**) Schematic structures of TGIF2a fusion constructs. TTGG, two threonines mutated to glycines. (**B**) Representative pictures of E12 primary cortical cultures transfected with different conditions at 3 days posttransfection, costained for PAX6, TBR2, and TUBB3. Magenta arrowheads for PAX6+TBR2-GFP+/GFP+ cells, yellow arrowheads for TBR2+PAX6+GFP+/GFP+ cells, and white arrowheads for TUBB3+GFP+/GFP+ cells. Scale bars, 50 μm. (**C**) AlphaFold prediction of TGIF2 structure, with DNA binding domain and repressor domain circled, and two mitogen-activated protein kinase (MAPK) sites indicated. (**D** and **E**) Quantifications of PAX6+GFP+/GFP+ and TUBB3+GFP+/GFP+ in transfected E12 culture at 3 days posttransfection with TGIF2a_KRAB, TGIF2a_VP64, and TGIF2a^TTGG^ constructs; mean + SD. *N* = 3 to 9 pools of embryos. Ordinary one-way ANOVA with Dunnett’s multiple-comparison test. n.s., not significant. (**F**) Schematic drawing of immunoprecipitation–mass spectrometry (IP-MS) experiment in P19 cells. h, hours. (**G**) STRING analysis of interactors of TGIF2a with label-free quantification (LFQ) intensity more than threefold compared to GFP control.

To determine whether TGIF2 acts as a repressor in NSCs, we performed sequence alignment with its paralog TGIF1, which is better characterized (*22*). This alignment revealed a conserved SIN3A-interacting domain (SID) in TGIF2, previously shown to interact with the SIN3A in TGIF1 and suggested to maintain pluripotency by repression (Fig. 3, A and C) (*22*, *23*).

To test the functional relevance of this domain, we generated TGIF2a fusion constructs replacing the SID with either a more potent repressor domain, Krüppel-associated box (KRAB), or an activator domain, tetrameric repeat of herpes simplex viral protein 16 (VP64) (Fig. 3A). TGIF2a-KRAB OE in E12 cortical cell cultures resulted in the same but even stronger phenotype than wild-type TGIF2a, showing a significantly higher proportion of PAX6+ NSCs (32.8%) compared to wild-type TGIF2a (17.2%) and control (8.7%) at 3 days posttransfection (Fig. 3, B and D). This was accompanied by a substantial reduction in the neuronal population in the TGIF2a-KRAB condition (Fig. 3E). Conversely, TGIF2a-VP64 OE led to a decrease in NSCs (Fig. 3, C and D), with more than 90% of cells differentiating into neurons (Fig. 3E). To determine whether it is the SID domain that is necessary to prevent premature differentiation and maintain NSC identity, we introduced a mutation in the middle of SID (fig. S4E). This mutant form shows a phenotype mimicking TGIF2 KD—more neurons at 3 days posttransfection (fig. S4, F to H).

Because phosphorylation has been reported to regulate TGIF2 in other systems (e.g., cancer) (*17*) and its phosphorylation sites seem to be a linker connecting the homeodomain and SID (Fig. 3A), we next investigated its relevance in neurogenesis. We generated a phospho-deficient TGIF2a mutant by substituting two mitogen-activated protein kinase (MAPK) threonine residues with glycine (TGIF2a^TTGG^) (Fig. 3, A and B). OE of phospho-resistant TGIF2 in E12 cortical cultures did not affect NSC maintenance or neuronal differentiation (Fig. 3, C to E), suggesting that TGIF2a function requires its phosphorylated form to repress differentiation and promote NSC maintenance.

Collectively, these findings demonstrate that TGIF2 exerts its transcriptional repressive function through a conserved SID, which normally mediates interaction with the SIN3A–histone deacetylase (HDAC) complex (*22*), and that this function is dependent on TGIF2’s phosphorylation state. These properties are critical for its role in maintaining NSC identity and repressing premature neuronal differentiation.

### TGIF2 interacts with HDAC1/2 and SIN3 corepressor complex

To uncover molecular partners that mediate TGIF2’s transcriptional repression, we performed coimmunoprecipitation, followed by mass spectrometry (co-IP–MS) using the triple-flag–tagged TGIF2a expressed in P19 cells (Fig. 3F). In two independent biological replicates, we identified high-confidence interactors by comparing TGIF2a pulldowns to GFP controls [label-free quantification (LFQ) intensity ratio > 3; table S8]. Notably, TGIF2a robustly associated with core components of the SIN3A corepressor complex, including HDAC1/2 and retinoblastoma binding protein 4 and 7 (RBBP4/7)—key mediators of transcriptional repression via histone deacetylation (Fig. 3G) (*18*). In addition, TGIF2a interacted with nuclear lamina–associated proteins such as barrier to autointegration factor 1 (BANF1) and thymopoietin (TMPO) (also known as lamin-associated polypeptide 2), which contribute to gene silencing through chromatin tethering to the nuclear periphery (*24*).

Beyond chromatin regulators, TGIF2a also pulled down proteins involved in DNA replication and cell cycle [replication protein A 1, 2, and 3 (RPA1/2/3)], as well as metabolic control [poly(ADP-ribose) polymerase 1 (PARP1) and single-stranded DNA binding protein 1 (SSBP1)], suggesting broader regulatory roles (Fig. 3G). To verify that TGIF2a physically interacts with the SIN3A-HDAC complex as shown before (*18*), we performed IP–Western blot experiments. Using an anti-Flag antibody to immunoprecipitate TGIF2, we could pull down HDAC1, HDAC2, and SIN3A but not in the immunoglobulin G (IgG) control or in cells transfected with the GFP control plasmid (fig. S4I). These data thus provide molecular evidence in support of TGIF2’s repressive function by interacting with the SIN3A/HDAC complex.

### TGIF2 OE in vivo expands the NSC pool and delays neuronal positioning

**Fig. 4.**
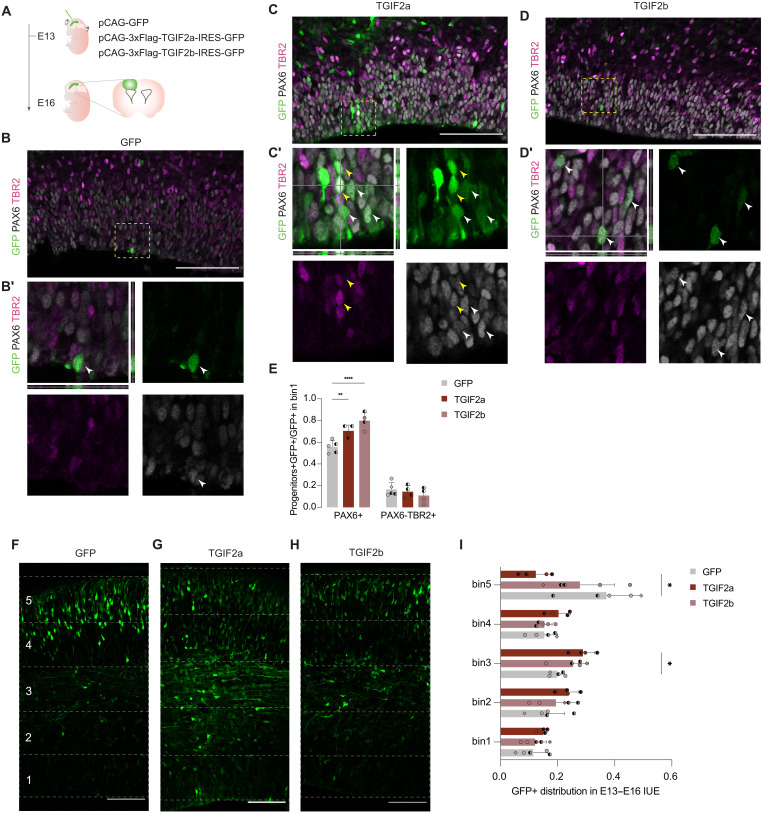
TGIF2 OE in vivo retains NSCs and immature neurons. (**A**) Experimental scheme of IUE, including used plasmids. (**B** to **D**) Sections of electroporated cortices stained with PAX6 and TBR2, of which insets are to show large magnifications with orthogonal views in (B′) to (D′). Scale bars, 100 μm. (**E**) Quantifications of PAX6+GFP+/GFP+ and PAX6-TBR2+GFP+/GFP+ cells in bin; mean ± SD. *N* = 4 to 5 embryos from at least two different mothers. Different symbols indicate different mothers. Ordinary two-way ANOVA with Dunnett’s multiple-comparison test. **P < 0.01 and ****P < 0.0001. (**F** to **H**) Representative images of CTX 3 days after electroporation with each condition in GFP. Scale bars, 100 μm. Dashed lines indicate the five equal bins. (**I**) Quantification of GFP+ cell distribution per bin at 3 days postelectroporation; mean ± SD. *N* = 4 to 5 embryos from at least two different mothers. Different symbols indicate different mothers. Multiple unpaired *t* tests with 5% FDR. *q < 0.05.

Given the evidence of TGIF2 promoting NSC fate via its repressive function in vitro, we aimed to determine whether this was also the case in vivo and whether it would affect neurogenic primed gene expression. Toward this aim, we used the same OE constructs for in utero electroporation (IUE) into the cerebral cortex at E13 and analyzed the cortices 3 days post-IUE (Fig. 4A).

To determine the effect of TGIF2 on NSCs and NPCs, we performed immunostaining using PAX6 for labeling NSCs and TBR2 for labeling NPCs (Fig. 4, B to D). Both TGIF2 constructs resulted in a significant enrichment of PAX6+ NSCs, with no differences observed for TBR2+ cells (Fig. 4E), suggesting that TGIF2 favors NSC fate. Most PAX6+ cells were located in the ventricular zone (bin1) after TGIF2 OE similar to the control, and no ectopic PAX6+ or TBR2+ cells were detected under the OE conditions. To assess proliferation, we examined the mitotic marker phospho-histone 3 (pH3), which revealed a more than twofold increase in proliferating pH3+/GFP+ cells under TGIF2 OE, with TGIF2b isoform showing a stronger and significant effect (fig. S5, A and B).

To determine whether cells overexpressing TGIF2 remain stuck in the ventricular zones, we examined their position by dividing the cortical thickness into five bins, with bin1 being at the ventricle (Fig. 4, F to H). While cells were capable reaching the cortical plate, we observed a decrease of mature neurons in the cortical plate (bin5) upon TGIF2a OE (Fig. 4I). We also noted increased cell proportions in bin3 (Fig. 4I), which contained young neurons labeled with neuronal differentiation factor 2 (NEUROD2) (fig. S5, C to F). The two isoforms differed in the size of the effect, with TGIF2b affecting proliferation stronger and TGIF2a affecting neuronal differentiation and positioning more. These results demonstrate that TGIF2 favors NSCs and proliferation while delaying neuronal differentiation also in vivo.

### TGIF2 target genes include neuronal differentiation and neurogenic primed genes

**Fig. 5.**
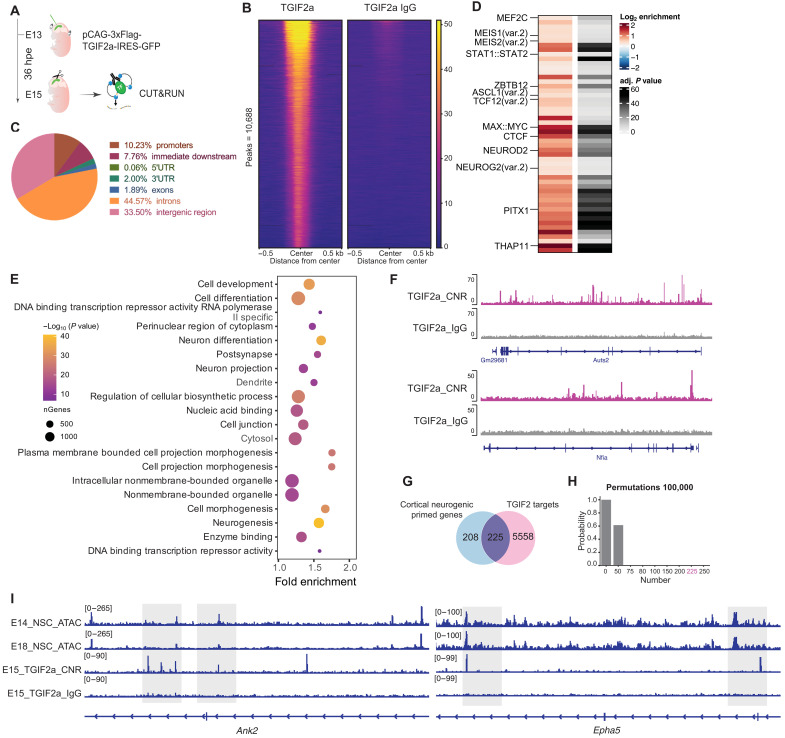
TGIF2 binds to neural differentiation and neurogenic primed genes. (**A**) Schematic drawing of experimental procedures of CUT&RUN. hpe, hours postelectroporation. (**B**) Enrichment heatmap of TGIF2a peaks and its corresponding IgG control, centered at the middle of the peaks. (**C**) Pie chart of genomic distribution of TGIF2a peaks. 3′UTR, 3′ untranslated region. (**D**) monaLisa motif enrichment analysis of TGIF2a peaks. adj., adjusted. (**E**) Top 20 terms from GO term enrichment analysis of annotated genes. (**F**) Peak examples with bigwig profiles exported from Integrative Genomics Viewer IGV (*83*). (**G**) Venn diagram of TGIF2a targets and CNP genes. (**H**) Permutation test with 100,000 trials to identify the possibility of equal-sized random gene sets to overlap with the CNP genes. (**I**) Examples of ATAC-seq profiles of neurogenic primed genes with TGIF2a CUT&RUN peaks exported from IGV (*83*). CNR, CUT&RUN.

To identify the genomic targets through which TGIF2 mediates its effects, we performed Cleavage under targets and release using nuclease (CUT&RUN), focusing on the TGIF2a isoform. The flag-tagged TGIF2a-OE construct as described above was electroporated into the cortex at E13, followed by CUT&RUN 36 hours later (Fig. 5A).

This experiment revealed 10,688 TGIF2a-bound peaks (Fig. 5B), predominantly located in intronic (44.6%) and intergenic regions (33.5%), suggesting a preference for regulatory elements over promoter regions (10.2%) (Fig. 5C). Motif enrichment analysis identified binding motifs for several key TFs involved in neurogenesis and neuronal differentiation, including achaete-scute family BHLH transcription factor 1 (ASCL1), NEUROD2, NEUROG2, myeloid ecotropic viral integration site 1 and 2 (MEIS1/2), and MYC (Fig. 5D), suggesting possible competition or cooperation at shared regulatory sites. While the canonical TGIF2 motif was not among the top enriched, motif scanning confirmed 3176 occurrences of its known binding sequence within the peak set (*P* < 0.001; table S9).

Annotation of the nearest genes associated with the peaks yielded 5783 target genes (table S9). GO analysis of these targets showed significant enrichment for neuronal differentiation processes supporting cell migration and synaptic maturation, including “neurogenesis,” “postsynapse,” “dendrite,” and “cell projection morphogenesis” (Fig. 5E). TGIF2 also targeted numerous RNA binding and splicing factors (e.g., *Stau1/2*, *Pum1/2*, *Ptbp2*, and *Snrnps*) and signaling mediators such as *Tle4, Tcf7l1*, and *Smad4*. Further analysis using Genomic Regions Enrichment of Annotations Tool (GREAT) revealed genes with particularly dense TGIF2 binding, such as *Auts2* and *Nfia*, each associated with approximately 20 intragenic peaks (Fig. 5F, fig. S6A, and table S9). These high-density targets were enriched in categories including “H4 histone acetyltransferase complex” (*Kansl1*, *Epc1*, and *Mllt3*), “growth cone” (*Dcc*, *Auts2*, and *Myh10*), and “chromatin” (*Brd4*, *Smarcc1*, and *Arid1b*) (fig. S6B), pointing to a broader role for TGIF2 in regulating chromatin remodeling and neuronal differentiation.

A total of 225 of the 433 CNP genes (51.9%) was directly bound by TGIF2 (Fig. 5G), indicating a substantial overlap between TGIF2 binding sites and accessible chromatin regions of primed genes in NSCs. To assess the statistical significance of this overlap, we generated 100,000 random sets of genes not bound by TGIF2a (equal in size to the TGIF2 target set) and compared their overlap with the CNPs. None of the permutations exceeded 77 overlapping genes (Fig. 5H), highlighting the specificity and significance of TGIF2 binding to them. We further investigated whether the notion of a gene as primed and/or its binding status by TGIF2 influences chromatin openness. Peaks in proximity to primed, TGIF2-bound genes showed significantly decreased accessibility compared to all other groups of peaks. These data are thus aligned with the concept that TGIF2 not only represses its targets but also reduces chromatin accessibility (fig. S6C). Together, these data establish TGIF2 as a key regulator of neurogenic priming and neuronal differentiation, acting through direct binding to primed chromatin regions of neuronal genes and regulatory hubs that orchestrate NSC identity and maturation.

### TGIF2 OE slows down neuronal differentiation shown by single-cell RNA-seq

**Fig. 6.**
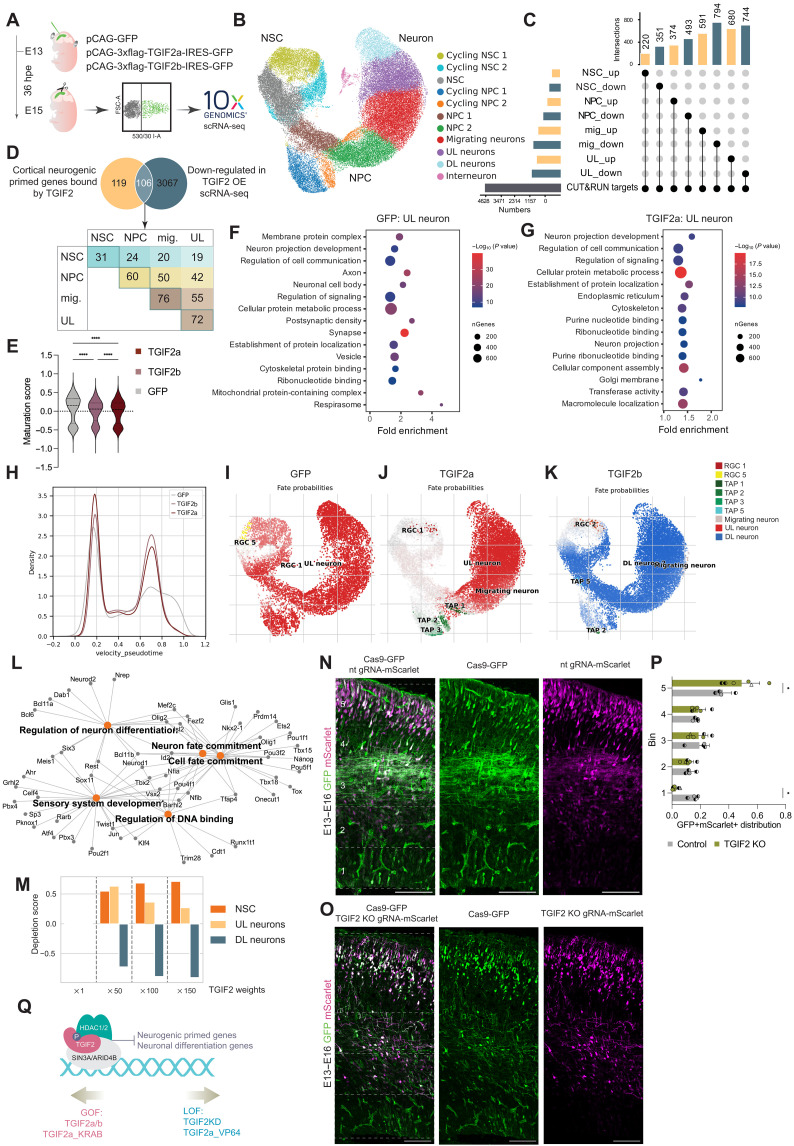
TGIF2 OE slows differentiation shown by scRNA-seq, and CRISPR knockout of TGIF2 promotes differentiation. (**A**) Schematic drawing of scRNA-seq experimental procedures and used plasmids. (**B**) UMAP projection with each cluster annotated with corresponding cell type. (**C**) UpSet plot overlapping TGIF2 CUT&RUN targets and DEGs in scRNA-seq between TGIF2a and GFP per cell type. (**D**) Venn diagram and intersection table of CNP genes bound by TGIF2 and down-regulated genes in scRNA-seq across different cell types. mig., migrating neurons. (**E**) Violin plot of maturation score per condition. Kruskal-Wallis test with Dunn’s multiple-comparison test. (**F** and **G**) Top 15 terms of GO term enrichment analysis of DEGs in upper layer (UL) neurons between GFP and TGIF2a. (**H**) Cell density plot along velocity pseudotime. (**I** to **K**) Fate probability maps from CellRank (*26*, *27*) analysis. RGC, radial glial cells; TAP, transit-amplifying progenitors. (**L**) Gene regulatory network (GRN) built by CellOracle (*76*), representing negatively regulated TFs by TGIF2a and associated GO terms. (**M**) Weighted simulations by RegVelo (*28*) for TGIF2a-OE effect on cell fate bias. (**N** and **O**) Representative pictures of CRISPR knockout (KO) experiment of TGIF2 by IUE, with Cas9-GFP plasmid in green and gRNA-mScarlet plasmid in magenta. Scale bars, 100 μm. (**P**) Quantification of GFP+mScarlet+ cell distribution per bin; mean + SD. *N* = 4 to 6 embryos from at least two mothers. Ordinary two-way ANOVA test with Sidak’s multiple-comparison test. *P < 0.05. (**Q**) Scheme of molecular mechanisms of TGIF2: When TGIF2 is phosphorylated, it is able to interact with SIN3A complex including AT-rich interaction domain 4B (ARID4B) and HDAC1/2, which together repress neurogenic primed and neuronal differentiation genes.

To elucidate the transcriptomic changes underlying TGIF2-mediated maintenance of NSC identity, we performed single-cell RNA-seq (scRNA-seq) on GFP+ cells isolated 36 hours after IUE via fluorescence-activated cell sorting (FACS; Fig. 6A). After quality control, 51,392 cells were retained for analysis. Dimensionality reduction via uniform manifold approximation and projection (UMAP) showed consistent overlap among conditions and replicates (fig. S6D). Cell clusters were identified via the Leiden algorithm (fig. S6E) and annotated on the basis of marker gene expression (Fig. 6B and fig. S6G). For instance, we identified NSCs, marked by *Pax6*, *Sox2*, and the radial glia marker *Fabp7*, and NPCs, marked by expression of *Eomes*, *Neurog2*, and *Elavl2* (fig. S6G). Cell cycle phases were inferred through cell cycle marker genes to identify cycling cell populations (fig. S6F).

Comparing TGIF2 expression levels between GFP control (representing endogenous TGIF2 levels) and TGIF2 conditions revealed that TGIF2 OE levels were mild in the stem cell cluster of radial glial cells (1.2 to 1.5×). Baseline expression levels were lower in migrating and differentiating neurons, and hence OE was higher in fold change but not reaching the levels of expression in NSCs or NPCs (fig. S6H). DEG analysis revealed a global down-regulation of transcripts in TGIF2-OE cells across cell types (Fig. 6C), many of which were direct TGIF2 targets identified by CUT&RUN, highlighting its transcriptional repressor function in vivo. Notably, approximately half of the CNPs directly bound by TGIF2 were down-regulated (Fig. 6D), including *Stmn2*, *Epha3*, and *Rorb*, reinforcing TGIF2’s major role in repressing primed neurogenic gene expression.

To quantitatively assess the impact of TGIF2 on neuronal maturation in vivo, we computed a maturation score based on the average expression of genes associated with neuronal differentiation and maturation (see Materials and Methods). TGIF2-OE cells displayed significantly lower maturation scores, with TGIF2a having a stronger effect than TGIF2b (Fig. 6E). In TGIF2a-OE NSCs, GO term analysis of down-regulated genes revealed significant enrichment in “mitotic cell cycle” and “neuron differentiation” (fig. S6I). Down-regulated genes associated with mitotic cell cycle include *Cdc45*, *Cdk1*, and *Ube2c*, suggesting insufficiency in G_2_-M transition and progression. This aligns with observed changes in cell cycle phase distributions, where TGIF2a OE promoted overall cell cycle length shown by time-lapse live imaging in E12 cortical cultures, as well as a significant enrichment in S and G_2_-M phases (fig. S7). In the upper layer (UL) neuron cluster, GO terms under both control and TGIF2a conditions showed general terms of neurogenesis (e.g., “neuron projection development”), but only control cells showed enrichment for more mature features such as “axon” and “postsynaptic density” (Fig. 6, F and G), suggesting that TGIF2a slows terminal neuronal maturation.

To further investigate the dynamics of differentiation, we performed pseudotime trajectory analysis using RNA velocity (*25*). Cells overexpressing TGIF2 remained predominantly in early differentiation states, whereas control cells progressed further along the neurogenic trajectory (Fig. 6H and fig. S8A). This delay was particularly evident in NPC2 and postmitotic neuron populations (fig. S8B). CellRank-based fate mapping identified 13 macrostates (fig. S8, C to E) (*26*, *27*). TGIF2a OE delayed the assignment of UL neuron fates and maintained cells more in NSC states (Fig. 6, I and J), underscoring TGIF2’s key role in modulating the tempo of fate transitions in cortical neurogenesis. The OE of the shorter isoform TGIF2b also delayed differentiation, maintaining some cells in NSC states, although the effect was slightly less pronounced than with TGIF2a (Fig. 6K). Unexpectedly, there was no UL neuronal fate being predicted in TGIF2b condition but only deep layer neuronal (DL neuron) fate (Fig. 6K and fig. S8E). Collectively, these analyses unbiasedly confirmed that TGIF2 OE down-regulates expression of neuronal differentiation genes, as well as mitotic cell cycle genes in NSCs, thereby maintaining cells in progenitor states, reminiscent of our findings based on immunostainings in vivo (Fig. 4) and in vitro (Fig. 2).

To uncover the underlying regulatory network, we used RegVelo (*28*), which integrated our E14 cortical NSC ATAC-seq data and TGIF2a CUT&RUN data to construct a priori gene regulatory network (GRN) and infer dynamics from the scRNA-seq control dataset (Fig. 6L). This GRN revealed a network of targets negatively regulated by TGIF2a and highlighted “neuron fate commitment” and neuron differentiation as key regulated terms. Among these RegVelo-refined and negatively regulated targets, Fez family zinc finger 2 (FEZF2) and B-cell lymphoma/leukemia 11B (BCL11B) are two critical TFs for DL neuron fate, suggesting that TGIF2a may directly repress DL neuron fate. When we applied weighted simulations in RegVelo to mimic TGIF2a OE, TGIF2a weights promoted NSC and UL neuron fates at the expense of DL neuron production (Fig. 6M). In addition, the more added weights we simulated, the bigger the enrichment in NSC fate, resonating with the phenotype in vitro and in vivo (Figs. 2 and 4).

Collectively, our single-cell transcriptomic and trajectory analyses reveal that TGIF2 functions as a transcriptional repressor that delays neuronal maturation by down-regulating neurogenic primed and differentiation genes. By broadly repressing these programs across neural stem and progenitor populations, TGIF2 sustains cells in an immature, undifferentiated state.

### CRISPR-mediated knockout of TGIF2 promotes neuronal differentiation

To functionally validate the role of endogenous TGIF2 in vivo, we performed CRISPR-mediated knockout (KO) of *Tgif2* via IUE (Fig. 6, N and O). KO efficiency was validated first by reverse transcription quantitative polymerase chain reaction (RT-qPCR) in N2A cells stably expressing Cas9, transfected with two separate candidate guide RNAs (gRNAs) targeting exon 2 of *Tgif2* (see Materials and Methods), which confirmed efficient KD of *Tgif2* while sparing *Tgif1* (fig. S9A). To additionally validate the effectiveness of the chosen gRNA1 at the protein level, we overexpressed TGIF2a and TGIF2b in N2A cells and cotransfected them with the Cas9-GFP plasmid used for the in vivo experiments and an mScarlet vector either with a non-targeting (nt) gRNA or with gRNA1 against Tgif2. The overexpression plasmids contain the targeted protospacer adjacent motif sequence, thus the ribonucleoprotein complex is functional. We found a complete KO of TGIF2a, while TGIF2b was only reduced to half, at the protein level (fig. S9B). For in vivo experiments, Cas9-GFP was co-electroporated with mScarlet-gRNA1 at E13, and brains were analyzed at E16.

A significantly higher proportion of GFP+ cells under the KO condition migrated into the cortical plate compared to controls, with very few cells remaining in the ventricular zone (Fig. 6P), exhibiting a significant reduction of cells expressing the NSC marker PAX6 (fig. S9, C and D). Thus, loss of TGIF2 promotes neuronal differentiation not only in vitro but also in vivo. These findings further corroborate the concept that TGIF2 acts as a molecular brake on neuronal differentiation.

To explore the effects of TGIF2 KO at the transcriptional level, we performed RNA-seq of NSCs after *Tgif2* KO in vivo (fig. S9E). More specifically, a Cas9-GFP plasmid was co-electroporated with mScarlet-gRNA1 against *Tgif2* at E13 (fig. S9, A and B, for gRNA validation), and GFP+mScarlet+Prominin1+ NSCs were isolated by FACS 48 hours after. SMARTseq2 low-input libraries were made and sequenced. We found 2045 DEGs at FDR of 5% of which 1682 were up-regulated and 363 were down-regulated, highlighting the main role of TGIF2 as a repressor also in vivo (fig. S9F and table S10). Gene set enrichment analysis of the transcripts up-regulated under the KO condition showed enrichments not only for signaling terms, immune processes, or genes related to migration but also for “positive regulation of gliogenesis,” with derepression of, e.g., *Egfr* (*29*), *Tnf* (*30*), *Stat3* (*31*), and *Id3* (*32*). These data thus suggest that TGIF2 is required to safeguard NSCs against not only neuronal but also glial differentiation. These data therefore align with the in vitro findings that TGIF2 regulates NSC maintenance. Conversely, however, not all neurogenic genes were derepressed, but some were further down-regulated with GO terms refer to “neuron fate specification” and axonogenesis (fig. S9G and table S10). These surprising enrichments are probably explained by a significant up-regulation of Tgif1 at the KO condition (log_2_ fold change = 1.76, FDR = 0.002; fig. S9H). As it is well known from work with KO mice that TGIF1 compensates for loss of TGIF2 (*33*), also in the brain in vivo, it appears that the loss of *Tgif2* (fig. S9, B and H) triggered *Tgif1* up-regulation. As TGIF1 has a very similar binding motif to that of TGIF2 (*18*), it may partially compensate loss of TGIF2 in repressing some of its neurogenic priming targets.

The above findings with partial compensation prompted us to examine the centrality of TGIF2 in regulating the CNP genes. CNP transcripts were more highly expressed than non-CNP transcripts both under control, as described above (fig. S1F), and KO conditions (fig. S9I), validating their role as CNPs. We then tested the enrichment of CNP transcripts in the DEGs after Tgif2 KO. Among all DEGs (FDR: 10%), CNP transcripts are significantly overrepresented using a Fisher’s exact test and 10,000 permutations (fig. S9J; Fischer’s *P* value = 0.03, odds ratio = 1.29), highlighting the importance of TGIF2 (and partially TGIF1) in regulating these genes.

## DISCUSSION

Here, we provide a comprehensive database to assess neurogenic and gliogenic priming in the murine forebrain across regions by RNA-seq and ATAC-seq of NSCs and neurons, both for specific regions or in a region-independent manner. Isolating NSCs at the peak of neurogenesis and onset of gliogenesis, with one region continuing some degree of neurogenesis, allowed to identify panneurogenic TFs including TGIF2. The comparison of ATAC-seq with RNA-seq reveals genes in a primed state during neurogenesis by examining neuronal genes lowly expressed with accessible regions at E14, which become less accessible upon transition to a different lineage (e.g., gliogenesis). While assigning peaks to nearest genes comes with a risk of identifying false positives, most of these peaks were intragenic and hence reliable. This analysis revealed several candidates for neurogenic priming of which we demonstrate TGIF2 as one key regulator. Notably, RNA-seq of TGIF2 KO NSCs revealed also derepression of gliogenic factors, further strengthening the concept of TGIF2 maintaining NSC fate by repressing neurogenic and gliogenic lineage differentiation.

This dataset can also be used to identify novel regulators of gliogenic priming—either for oligodendrogliogenesis [by comparing to RNA datasets of oligodendrocytes such as (*34*)] that starts de novo in the cortex or for astrogliogenesis [by comparing to RNA-seq datasets of astrocytes such as (*35*)] that takes over at E18 across regions. Moreover, region-specific analysis can be performed to identify upstream regulators of primed genes for neuronal subtypes when focusing on the LGE dataset. For example, generation of medium spiny neurons and cortical interneurons also comes to an end in E18 LGE NSCs, and analyzing genes higher expressed in these neurons, but open in E14 LGE NSCs, while closing in E18 LGE NSCs, could lead to region-specific regulators of neuronal subtype priming. This will allow to identify molecular regulators of the neuronal subtype priming described before also in the cortex (*2*). To facilitate further exploration of this dataset, a Shiny App has been made publicly available https://mrichter23.github.io/priming_repository/.

Here, we focused on neurogenic priming and identified the repressive function of TGIF2 as key in regulating most of the primed neurogenic genes in cortex NSCs along with maintaining their fate. We showed TGIF2 as a key regulator of NSC maintenance and neuronal differentiation by LOF and GOF experiments both in vitro and in vivo. TGIF2 functions as a molecular “brake” on neurogenesis programs, actively gatekeeping NSC and later NPC states, thereby interfering with premature differentiation and fine-tuning the timing of cortical development. By integrating single-cell transcriptomics, CUT&RUN, proteomics, and functional assays of fusion and mutant proteins, we demonstrated that TGIF2 maintains NSC fate not through its canonical role in antagonizing TGFβ signaling (*36*) (although some TGFβ-related genes were derepressed upon KO of TGIF2 in NSCs) but mostly by repressing neuronal and also glial differentiation genes to maintain NSC fate.

Here, we considered primed neurogenic genes in NSCs as those expressed significantly higher in neurons but already exhibiting associated open chromatin and low-level expression in NSCs at E14. TGIF2 bound more than half of them. As it is a panneurogenic factor expressed not only in LGE and cortex NSCs but also throughout central nervous system regions (*37*), we would propose TGIF2 functions as one key regulator of neurogenic priming in a wider context. Supporting the general relevance of our data also across species, RNA-seq data from the human cortex also revealed that TGIF2 expression steeply declines at gliogenesis stages (post–conceptual week 20) (*38*). Thus, TGIF2 represents a novel regulator of NSC maintenance during neurogenesis. TGIF2-mediated transcriptional repression allows primed NSCs to remain poised for differentiation cues and respond in a timely manner during the dynamic changes in neurogenesis. This is also reflected by higher expression of the primed neuronal genes compared to the nonprimed ones in young neurons. By maintaining basal expression levels of neuronal differentiation genes, TGIF2 ensures that NSCs are primed for lineage commitment without undergoing premature differentiation.

In this regard, TGIF2 itself is regulated by signaling pathways, namely, MAPK/extracellular signal–regulated kinase (ERK) signaling–induced phosphorylation, as shown before in cancer cells (*17*, *39*). Mutating the two MAPK phosphorylation sites in TGIF2 completely abolished its ability to promote NSC fate. Proteomic analysis of human induced pluripotent stem cell-derived NSCs and neurons (*40*) revealed that TGIF2 is phosphorylated only in NSCs but not in neurons, while its total protein levels remain unchanged (fig. S9K). The activation of MAPK/ERK is required for NSC proliferation and has to decline for neuronal differentiation (*41*, *42*). ERK activity is also suggested to be a gating mechanism for neural differentiation, as inhibition of ERK induced precocious transcription of neural genes in spinal cord precursors (*43*). These findings suggest that TGIF2’s activity is developmentally regulated by endogenous signaling pathways, such as MAPK/ERK signaling (*17*, *39*), modulating TGIF2’s interaction with the SIN3A complex.

Notably, TGIF2 binding sites determined by CUT&RUN are enriched with motifs for proneural TFs, such as ASCL1, NEUROG2, and NEUROD2, which are known to promote neurogenesis and neuronal differentiation in both developmental and adult contexts (*44*–*48*). This suggests that TGIF2 sterically blocks the access of proneural TFs and/or occupies the gene loci targeted by these neurogenic TFs, thereby inhibiting premature neuronal differentiation. This interplay between TGIF2 and neurogenic TFs may serve as a checkpoint to ensure the proper timing of neural differentiation during cortical development. In addition, among the genes repressed by TGIF2, we observed significant regulation of the nuclear factor I (NFI) family of TFs, including *Nfia*, *Nfib*, and *Nfix*, which are known to function synergistically (*49*). Double KO of *Nfia* and *Nfib* has been shown to cause ventricular enlargement from progenitor proliferation and reduced neural differentiation (*50*), a phenotype resembling TGIF2 OE with increased neural stem/progenitor cells and delayed differentiation. In addition, *Nfi* factors are also involved in gliogenesis (*51*, *52*), in line with TGIF2 also repressing some gliogenic TFs to maintain NSCs by repressing early gliogenesis (*32*). This finding places TGIF2 upstream of NFI family members in the regulatory hierarchy, functioning as a negative regulator of neuronal and glial differentiation promoted by these TFs.

TGIF2 also regulates various chromatin factors and histone modifiers, including *Arid1b*, *Arid4b*, and the histone methyltransferases and demethylases *Setbp1*, *Kdm1a*, and *Kdm7a*. Histone modifications, such as histone H3 lysine 36 methylation and H3K4 methylation in the context of bivalent marks, have been implicated in establishing epigenetically primed and “poised” transcriptional states (*6*). Thus, TGIF2’s regulatory influence may extend beyond direct transcriptional repression, potentially contributing to neurogenic priming through additional epigenetic mechanisms. This is also corroborated by our finding that CNP genes bound by TGIF2 in E14 NSCs are also less open compared to others as revealed by ATAC-seq.

Interactome analysis further determined factors that cooperate with TGIF2 to mediate repression, such as SIN3A and nucleosome remodeling and deacetylase (NURD) repressor complexes. Functional assays using the TGIF2-KRAB and TGIF2-VP64 fusion proteins further reinforced its role as a transcriptional repressor, as shown before (*17*, *18*), and also confirmed its direct interaction with SIN3A and HDAC2. SIN3A, in particular, regulates diverse cellular processes such as cell cycle, differentiation, and development (*53*, *54*) and has been implicated in neurological disorders such as intellectual disability (*55*), as well as cancer progression (*56*, *57*), some of the previously described roles of TGIF2 (*17*, *36*). TGIF2 appears to guide the SIN3A complex to specific DNA targets, restricting the expression of primed genes and fine-tuning the transcriptional regulation of neurogenesis and neural differentiation.

TGIF2’s function in the developing nervous system differs significantly from its role in other tissues. Unlike its reported interactions with SMAD proteins to regulate TGFβ target genes in other contexts, TGIF2 was not found to interact with SMAD proteins in this study. In addition, while in many cancer cells, TGIF2 promotes epithelial-mesenchymal transition, e.g., in lung adenocarcinoma cells (*17*), it maintains the epithelial-like NSCs in the developing cortex as shown here. Therefore, TGIF2’s role in the nervous system exhibits significant mechanistic differences compared to cancer cells and endoderm-derived tissues, where it has been more extensively examined (*21*, *58*). It was never characterized in priming, and no major factors regulating transcriptional panneurogenic priming were previously known. In summary, our findings establish TGIF2 as one major regulator of maintaining NSC fate across regions, using transcriptional repression to ensure the precise timing of cortical development.

## MATERIALS AND METHODS

### RNA-seq and ATAC-seq library preparation

Wild-type C57BL/6J embryos at E14 and E18 were used for the RNA-seq experiments, with tissue of one litter per mother being pooled and considered one biological replicate. Brains were dissected in 1× Hanks’ balanced salt solution (HBSS; Gibco, catalog no. 14025) with 10 mM Hepes (Gibco, catalog no. 15630). Lateral cortex from the mediolateral to the cortex-LGE border and LGE without overlying ventrolateral cortex were dissected and centrifuged at 1000 rpm at 4°C for 5 min. Dissection buffer was aspirated, and tissue was enzymatically dissociated with 1 ml of 0.05% Trypsin/EDTA (Gibco, catalog no. 25300) for 15 min at 37°C. Digestion was inhibited by adding 2 ml of Dulbecco’s modified Eagle’s medium (DMEM; Gibco, catalog no. 61965) with 10% fetal bovine serum (FBS; PAN-Biotech, catalog no. P30-3302), and tissue was further mechanically dissociated with a fire-polished glass Pasteur pipette coated with DMEM + 10% FBS to obtain a single-cell suspension. The suspension was centrifuged at 300*g* at 4°C for 5 min, the supernatant was aspirated, and the cells resuspended in 1× Staining Solution [1× HBSS, 1% glucose, 1 M Hepes, 1% FBS, 0.1% (w/v) NaN_3_, 1 mM EDTA, and DMEM-F12]. The cell suspension was stained with the preabsorbed antibody mCD133–phycoerythrin (PE) at 1:500 dilution [Anti-Mouse-CD133-PE (13A4), eBioscience/Invitrogen, catalog no. 12-1331-82]. A corresponding isotype control antibody (Mouse IgM-APC, Miltenyi Biotec, catalog no. 130-093-176) was added to an isotype control sample in the same dilution. Cells were incubated at 4°C in the dark for 25 min, and then 4′,6-diamidino-2-phenylindole (DAPI) [1:1000 dilution of stock (1 mg/ml); Sigma-Aldrich, catalog no. D9542] was added, followed by another 5 min of incubation. To wash the cells, the suspension was filled up to 10 ml with phosphate-buffered saline (PBS; Gibco, catalog no. 14190) and centrifuged at 300*g* at 4°C for 5 min. Cells were resuspended in PBS and filtered through a cell strainer (pluriStrainer Mini 40 μm, pluriSelect, catalog no. 43-10040-60) into suitable sample tubes (Falcon Round Bottom Polypropylene Test Tubes with Cap, Falcon, catalog no. 352063).

Cells were sorted on a FACSAria III Cell Sorter (BD Biosciences) with the FACSDiva software (version 6.1.3, BD Biosciences). To separate the populations, the first gate was set to separate small debris [low forward scatter (FSC)] and dead or damaged cells, which were DAPI+ (high 450/40 signal). The second gate was set to remove doublets or cell aggregates by FSC-area/FSC-width. The third gate separated the stained populations by the laser lines 582/15 for PE, with the gate set so that maximum of 0.1% of the parent population in the isotype control was detected as single or double positive. Sorted cells were collected in PBS and centrifuged at 300*g* at 4°C for 10 min. The supernatant was aspirated, and cells were immediately lysed in RNA extraction buffer.

For the RNA-seq libraries, total RNA extraction was performed with the PicoPure RNA Isolation Kit (Applied Biosystems, catalog no. KIT0204) according to the manufacturer’s protocol with on-column deoxyribonuclease (DNase) digestion (On-Column DNase I digestion set, Sigma-Aldrich, catalog no. DNASE70). RNA concentration and quality were evaluated on the Bioanalyzer (Model 2100, Agilent) using the RNA 6000 Pico Kit (Agilent, catalog no. 5067-1513) according to the manufacturer’s protocol. Samples with an RNA Integrity number (RIN) < 8.0 were excluded from library preparation. First-strand cDNA was prepared from 2 ng of RNA per sample with the SMART-Seq v4 Ultra Low Input RNA Kit for Sequencing (Takara/Clontech, catalog no. 634897) according to the manufacturer’s instructions. Number of amplification cycles for each sample was determined with a side qRT-PCR reaction performed after the first 4 amplification cycles to avoid overamplification bias. With this, the number of required total amplification cycles for each sample corresponded to the cycle number at ¼ of the maximum fluorescence signal (normalized reporter).

The amplified cDNA was purified using AMPure XP magnetic beads (Beckmann Coulter, catalog no. QT650) and quality and quantity analyzed by Bioanalyzer (High Sensitivity DNA Kit, Agilent, catalog no. 5067-4626) and Qubit Assay (Qubit dsDNA HS Assay Kit and tubes, Invitrogen, catalog nos. Q32854/Q32856). Purified cDNA was fragmented by ultrasonic shearing on the Covaris AFA S220 system using corresponding tubes (microtube AFA Fiber Pre-Slit Snap-Cap 6x16mm, Covaris, catalog no. 520045), resulting in ~200– to 500–base pair (bp)–long fragments that were purified by ethanol precipitation. Samples were evaluated again on the Bioanalyzer (HS DNA assay) before proceeding to the library preparation with the MicroPlex Library Preparation Kit v2 (Diagenode, catalog no. C05010014) according to the manufacturer’s instructions, using 10 ng of cDNA per sample. Following the library amplification, cDNA concentration was verified by Qubit assay, and the libraries were purified over AMPure XP magnetic beads. Quality and quantity of these final libraries were evaluated by Bioanalyzer HS DNA assay, and samples were multiplexed at 5 nM each. Next-generation sequencing was performed on an Illumina HiSeq 4000 system with 100-bp paired-end deep sequencing.

For the ATAC-seq libraries, nuclei were isolated from 50,000 cells using a cell lysis buffer containing 1 M tris-HCl, 5 M NaCl, 1 M MgCl_2_, 10% NP-40, 10% Tween 20, and 2% digitonin. They were subsequently resuspended in transposition mixture containing the transposase enzyme, 2% digitonin, and 10% Tween 20 and incubated for 30 min at 37°C. After the incubation, the samples were immediately put on ice, and DNA was purified with the MinElute Reaction Cleanup kit (QIAGEN, #28204). The transposed DNA was PCR amplified with the NEBNext High-Fidelity 2x PCR Master Mix (NEB, #M0541S). The number of cycles was determined with a qRT-PCR using the SensiMix SYBR No-ROX 2x Master Mix (Bioline, #QT650) as the number of cycles that corresponds to ¼ of the maximum fluorescence. The amplified libraries were purified, and the quality was assessed with a High Sensitivity DNA Chip (Agilent, #5067-4626). Size selection between 100 and 600 bp was performed with AMPure beads (Beckmann Coulter, #A63881), and libraries were pooled and sequenced on an Illumina HiSeq 4000 system with 100-bp paired-end deep sequencing.

### RNA-seq analysis

The quality of sequencing data was analyzed with FastQC v0.11.4 (*59*), and adapter trimming was performed with cutadapt v1.11 (*60*). Reads were aligned with the mouse reference genome (mm39) using STAR v2.6.0a (*61*). Afterward, reads were deduplicated, and gene expression was quantified with featureCounts v1.6.4 (*62*). The subsequent analysis was performed in R version 4.4.1 (*63*). Genes with less than 10 counts across all samples were excluded. The expression data were normalized and transformed using the vst function of DESeq2 v1.44.0 (*64*) for plotting and outliers’ analysis. To identify outliers, we performed a PCA. Samples with a distance of more than 2.5 SDs from the mean in the first principal component were excluded (no outliers were detected). Differential expression (DE) analysis was performed using DESeq2 v1.44.0 (*64*). DE analysis for each comparison was done separately. We tested for DE with DESeq2 using the Wald test and reported the genes with an FDR below 1% in the NSC maintenance analysis and below 5% in the priming analysis, respectively, as significant. In the priming analysis, PANTHER v19.0 (https://pantherdb.org/about.jsp) was used for GO analysis with all genes up-regulated in neurons versus NSCs as background (5835 genes). Data were plotted using the ggplot2 v3.5.1 package in R and the matplotlib v3.5.3 and seaborn v0.12.2 packages in Python.

For identification of TFs, we used the GO term GO:0140110. For identification of chromatin remodelers, we used the following GO terms: GO:0034724, GO:0031497, GO:0031498, GO:0034401, GO:0006338, GO:0016569, GO:0090202, GO:0070828, GO:0034728, and GO:0006342.

### ATAC-seq analysis

FastQC was used to assess initial data quality. Reads were trimmed using trim-galore with parameter --nextera after contamination of Nextera transposase sequence was found in the reads. After trimming, reads were aligned to mm39 reference genome using bwa-mem. The ATACseqQC R-package tutorial was followed to assess data quality and to shift reads by 5 bp as recommended (*65*). For each individual sample, peaks were called using MACS3 with parameters -f BAMPE -g mm -q 0.01 (*66*). Differential openness of peaks between either time points per tissue or tissues per time point was identified using DiffBind with parameter peakFormat = “narrow” when loading the samples. Homer was used to find motifs in the resulting differentially open peaks. Homer was also used for labeling the differential or consensus peaks by genes in proximity. Overlaps between peaks were identified by the function subsetByOverlap. To investigate whether the notion of a gene as primed and/or its binding status by TGIF2 changes chromatin accessibility, we used peaks that are closing in E18 and compared their openness level depending on whether the gene in proximity is (i) neither primed nor bound by TGIF2, (ii) primed but not bound by TGIF2, (iii) not primed but bound by TGIF2, or (iv) primed and bound by TGIF2.

### Plasmids

TGIF2 cDNA isoform plasmids were obtained from as a kind gift from previously described (*21*). All plasmids for expression were cloned into a Gateway (Invitrogen) form of pCAG-IRES-GFP (kind gift of P. Malatesta) through pENTR1a vector. TGIF2 cDNA was amplified by PCR with primers containing triple FLAG sequence for inserting the FLAG tag at N terminus of TGIF2 and cloned into the pCAG plasmid via Gibson assembly. Short hairpin RNA (shRNA) plasmids were designed using Invitrogen BLOCK-iT RNA designer and ordered as oligos from Eurofins and then ligated to pENTR1a vector with a GFP reporter, which was finally cloned into a pCAG destination vector via Gateway LR clonase.

### Mice

The animals were housed in the Core Facility Animal Models, Biomedical Center Faculty of Medicine, LMU Munich. They were maintained under specific pathogen–free conditions and housed in groups of two to three animals in individually ventilated cage systems with a 12-hour/12-hour light/dark cycle. C57BL/6J mice (Charles River Laboratories, Sulzfeld, Germany) were used for this study, and all animals undergoing IUE were females aged between 3 and 6 months. E0 was designated as the day of vaginal plug detection. Mice had free access to water and standard rodent chow (Altromin, 1310M). Experimental procedures were performed in accordance with animal welfare policies and approved by the Government of Upper Bavaria (Germany), under the license numbers ROB-55.2-2532.Vet_02-20-77 and ROB-55.2-2532.Vet_02-25-32.

### Anesthesia

For surgical procedures, mice were anesthetized via intraperitoneal injection of a solution containing fentanyl (0.05 mg/kg), midazolam (5 mg/kg), and medetomidine (0.5 mg/kg). Anesthesia was terminated with a subcutaneous injection of a solution comprising buprenorphine (0.1 mg/kg), atipamezole (2.5 mg/kg), and flumazenil (0.5 mg/kg).

### In utero electroporation

Pregnant dams at E13 were anesthetized and operated on according to established procedures (*40*). Briefly, endotoxin-free plasmids at 0.5 to 0.7 μg/μl, controlled for molar ratio across conditions, were diluted in 0.9% NaCl and mixed with Fast Green FCF dye. Subsequently, 1 μl of this mixture was injected into the lateral ventricle of embryos at E13 within anesthetized C57BL/6J mice. Embryonic brains were harvested at 3 days postelectroporation and fixed using 4% paraformaldehyde (PFA) in 1× PBS for durations of 4 hours. Analysis involved embryos obtained from at least two female mice, with quantification carried out on two to three coronal sections from three to five embryos.

### Cell culture

Cerebral cortices from C57BL/6J E12 mouse embryos were dissected in ice-cold HBSS buffered with 10 mM Hepes (both from Life Technologies). Cells were enzymatically dissociated with 0.05% Trypsin and mechanically triturated with a Pasteur pipette to obtain a single-cell suspension. These cells were then seeded in poly-d-lysine–coated coverslips in 24-well plates at 350,000 to 500,000 cells per well in DMEM-GlutaMAX supplemented with 10% FBS and 1% penicillin-streptomycin (Pen/Strep) and incubated at 37°C with 5% CO_2_. After 24 hours, 2% B27-supplemented DMEM-GlutaMAX with 1% Pen/Strep were added at 1:1 ratio. Three or 7 days posttransfection, cells were fixed with 4% PFA for 10 min at room temperature.

For transfection experiments, cells were plated and allowed to adhere for 2 to 3 hours before transfection with either 0.5 to 0.7 μg of plasmids controlled for molar ratio or 25 nM siRNA Tgif2 mouse (ON-TARGETplus SMARTpool) using Lipofectamine 2000 following the manufacturer’s guidelines (Invitrogen). When shRNAs were cotransfected with GFP or TGIF2a-OE plasmids, equal molarity ratio was controlled.

### Immunohistochemistry and immunocytochemistry

Sections underwent triple washes with 1× PBS at room temperature before being incubated overnight at 4°C with primary antibody in a blocking solution, composed of 10% normal goat serum and 0.5% Triton-X 100 in 1× PBS. Cells were first incubated in blocking solution for 1 hour at room temperature, followed by overnight incubation with primary antibody. After triple wash with 1× PBS at room temperature, cells and sections were stained with secondary antibodies diluted in blocking solution for 1 hour at room temperature. Nuclei were visualized using DAPI (0.5 μg/ml; Sigma-Aldrich). Last, immunostained sections and cells were examined using a Zeiss confocal microscope. The list of antibodies used in the experiments is provided for reference. For Fig. 2, a sequential staining was performed to costain a mouse IgG1 PAX6 and a mouse IgG2b Tubulin3 antibodies. More specifically, the same protocol as outlined above was performed, but the secondary antibody washed a 4% PFA incubation was performed for 5 min at room temperature, which was followed by the same protocol as above including the blocking, but the Triton was substituted with Tween.

### scRNA-seq library prepration

Thirty-six hours after IUE, cortices were dissected in ice-cold HBSS buffered with 10 mM Hepes (both from Life Technologies) under florescent microscope to enrich for electroporated region. The cells were dissociated to arrive at single-cell suspension with Neural Tissue Dissociation Kit(P) (Milteny, #130–092-628) and red blood cell removal solution (Miltenyi Biotec, #130-094-183) following the manufacturer’s protocol. The cells were passed through a 40-μm cell strainer and placed on ice for FACS to further isolate electroporate cells. FACS sorting was performed at a FACSAria III (BD Biosciences) in FACSFlow sheath fluid (BD Biosciences), with a nozzle diameter of 100 μm. Debris and aggregated cells were gated out by forward and side scatter, respectively. Single cells were selected by FSC-W/FSC-A. Gating for GFP fluorescence was done using nonelectroporated cortices.

FACS-sorted cells were multiplexed using Cell Multiplexing Oligo Labeling and loaded onto 10x Chromium chip following Single Cell 3′ v3.1 (Dual Index) protocols with Feature Barcode technology for Cell Multiplexing (CG000388). The library was sequenced with one NovaSeq 6000 S2 flowcell to reach 30,000 reads per cell for gene expression library and 5000 reads per cell for multiplexing library, which was then aligned and demultiplexed using cellranger multi pipeline.

### scRNA-seq analysis

The analysis followed Scanpy’s (*67*) tutorial, starting with preprocessing of raw sequencing data to filter out low-quality cells (counts per cell = 1100 to 33,000, minimal genes per cell = 700) with high mitochondrial content (5% cutoff), followed by log transformation normalization. Dimensionality reduction using PCA and UMAP was performed to visualize cell-to-cell relationships. Leiden clustering identified distinct cell populations based on gene expression profiles, and marker genes were determined to characterize each cluster’s cell types. Maturation score included genes *Neurog2*, *Dcx*, *Tubb3*, *Elavl4*, *Map2*, *Stmn2*, *Rbfox3*, *Syt1*, *Nefl*, *Syn1*, *Syp*, *Camk2a*, and Bsn. DE between TGIF2a and GFP was analyzed using built-in “rank genes” function in Scanpy with Wilcoxon rank sum test, and associated GO term was analyzed using ShinyGO 0.80 (*68*). CellRank analysis based on RNA velocity was conducted following the CellRank’s tutorial (*26*, *27*).

### CUT&RUN and library preparation

Electroporated embryos underwent the same procedure as described in scRNA-seq section until before FACS. CUT&RUN was performed using CUT&RUN assay kit (Cell Signaling Technology, 86652) according to the manufacturer’s instructions. Briefly, 250,000 cells per reaction were collected and bound to Concanavalin A magnetic beads. Cells were permeabilized and incubated with 1 μg of primary antibody against FLAG [DYKDDDDK Tag (D6W5B), rabbit, Cell Signaling Technology] per sample overnight at 4°C. The rabbit (DA1E) mAb IgG XP Isotype Control antibody was used as IgG control. Subsequently, cells were incubated with pAG-MNase for 1 hour at 4°C. pAG-MNase was activated by adding calcium chloride and incubation at 4°C for 30 min. Stop buffer (Cell Signaling Technology) was added to each sample to stop the reaction. DNA was purified using phenol/chloroform extraction and ethanol precipitation as described in the manufacturer’s protocol.

DNA sequencing libraries were generated using the SimpleChIP ChIP-seq DNA Library Prep Kit for Illumina (Cell Signaling Technology, 56795) and SimpleChIP ChIP-seq Multiplex Oligos for Illumina (Dual Index Primers, Cell Signaling Technology, 46538) following the manufacturer’s instructions specifically for CUT&RUN Assay kit protocol. Briefly, 5 ng of DNA was used for all CUT&RUN and IgG control samples. DNA ends were ligated with adaptors and amplified using PCR and Dual Index primers for Illumina (Cell Signaling Technology, 47538). All cleanup steps were performed with 1.1× volume of SPRIselect beads to increase the capture of smaller DNA fragments. Generated libraries were pooled and sequenced using 2 × 75 bp paired-end sequencing strategy on an Illumina NextSeq 550 sequencer.

### CUT&RUN analysis

Sequenced reads were aligned to the mm39 genome using Bowtie2 (*69*). Peak calling was performed using the MACS3 pipeline (*66*) with corresponding IgG control bam files, using *q* value of 0.01, and minimal fragment length: 100. An enrichment heatmap of the peaks was produced using deepTools’s computeMatrix function (*70*) on Galaxy platform (*71*). FIMO motif scanning was conducted on MEME Suite website using bed file of identified peaks (*72*). In addition, monaLisa was used for motif enrichment analysis on the identified peaks (*73*). The peaks were analyzed for genomic distribution with ChIPpeakAnno (*74*) and annotated using GREAT for single nearest gene within 250 kb (*75*). GO term enrichment analysis with ShinyGO 0.80 was conducted on the annotated genes (*68*).

### Gene regulatory dynamic analysis

RegVelo is an end-to-end deep generative model designed to infer cellular dynamics through coupled splicing dynamics and gene regulation (*28*). It requires users to define the prior GRN and allows the model to refine this network by improving the reconstruction of observed gene expression. Using a bulk ATAC-seq dataset, we followed CellOracle’s tutorial (*76*). First, we identified transcription start sites (TSS) using the get_tss_info function, which annotates each peak with its corresponding gene. Next, we scanned TF binding motifs in these peak regions using the tfi.scan function with an FDR of 0.02. Subsequently, we filtered motifs using the filter_motifs_by_score function with a threshold of 10. Last, we replaced the bulk ATAC-seq–derived TGIF2 targets with CUT&RUN-inferred target genes and incorporated this prior GRN for downstream RegVelo analysis.

We trained the RegVelo model with default parameters. To mimic OE effects, we manually perturbed the inferred gene regulation by multiplying TGIF2 downstream regulation weights by a specific factor to amplify the regulatory effects of TGIF2. We used four different values (0, 50, 100, and 150) and used RegVelo to predict the depletion scores (*28*) for defined terminal states, including NSCs, UL neurons, and DL neurons. RegVelo-inferred GRN targets were used for downstream gene functional analysis. We curated all negatively regulated genes inferred by RegVelo and applied the clusterProfiler package to perform GO enrichment analysis.

### Coimmunoprecipitation

For interactome analysis, P19 cells were seeded in 10-cm dishes for transfection when the cells reached 50% confluency. After 48 hours, cells were scraped on ice and lysed in nondenaturing lysis buffer [20 mM tris-HCl (pH 8.0), 137 mM NaCl, 1% NP-40, and 2 mM EDTA] containing cOmplete proteinase inhibitor. Lysates were incubated with DYKDDDDK Tag (D6W5B) FLAG rabbit antibody (Cell Signaling Technology) for 1 hour, followed by addition of Protein G Dynabeads for an additional 2 hours at 4°C with rotation. Following three washes with wash buffer [10 mM tris, (pH 7.4), 1 mM EDTA, 150 mM NaCl, and 1% NP-40], the immunoprecipitated lysates were boiled in 1× Laemmli buffer and subsequently stored at −80°C until MS analysis.

For co-IP–Western analysis, N2A cells (mouse neuroblastoma cells) were used. The cells were seeded in 10-cm dishes and transfected when they reached 65% confluency with 15 μg of DNA per dish. After 48 hours, cells were scraped and snap-frozen dry in dry ice and kept at –80°C. For the co-IP, cells were lysed in nondenaturing lysis buffer [20 mM tris-HCl (pH 8.0), 137 mM NaCl, 1% NP-40, and 2 mM EDTA] containing cOmplete proteinase inhibitor. Protein G Dynabeads were blocked in PBS with BSA (1 mg/ml) for 2 hours at 4°C on a rotator, while the lysates were incubated with DYKDDDDK Tag (D6W5B) FLAG rabbit antibody (Cell Signaling Technology) or a rabbit IgG isotype control antibody (Cell Signaling Technology, 66362S) for 2 hours at 4°C on a rotator. Following that, the cell lysates–antibody lysate was added on Protein G Dynabeads for an additional 2 hours at 4°C with rotation. Following three washes with wash buffer A [10 mM tris (pH 7.4), 1 mM EDTA, 150 mM NaCl, and 1% NP-40] and two washes with wash buffer B [10 mM tris (pH 7.4), 1 mM EDTA, and 150 mM NaCl], the immunoprecipitated lysates were boiled for 5 min at 95°C in 1× Laemmli buffer including β-mercaptoethanol and subsequently stored at −80°C until Western blot analysis. For Western blot analysis, co-IP pulldowns and input samples were supplemented with 2× loading buffer, boiled at 95°C for 5 min, and spun down at full speed for 5 min at 4°C. Protein lysates were run on an 8% gel and blotted on nitrocellulose membranes. The antibodies used for blotting were anti-HDAC1 (1:1000; Cell Signaling Technology, #2062S), anti-HDAC2 (1:1000; Cell Signaling Technology, #2540S), anti-SIN3A (1:1000; Life Technologies, #PA590691), anti-FLAG (1:10,000; Sigma-Aldrich, F1804), anti-rabbit horseradish peroxidase (HRP; Life Technologies, 31458), and anti-mouse HRP (Life Technologies, 31432).

### Mass spectrometry

The interactome samples were digested using a modified FASP procedure as described (*77*, *78*). Digested peptides were measured on a Q Exactive HF-X mass spectrometer (Thermo Fisher Scientific) online coupled to an UltiMate 300 RSLCnano (Thermo Fisher Scientific) as described (*79*). Generated raw files were quantitatively analyzed in the MaxQuant software (*80*) (MPI Martinsried, version 2.4.9.0), applying default settings and a minimum LFQ ratio count of 1, quantification on unique peptides with matching between runs for LFQ quantification (*81*). Searches for peptide identifications were performed in the integrated search engine Andromeda (*82*) with default settings, using the canonical SwissProt Mouse protein database including the described TGIF2 sequences. Results were filtered for contaminant hits, reverse hits, and “only identified by site” hits. LFQ intensity values in the filtered proteingroups list were used for enrichment ratio calculations.

### Time-lapse live imaging

E12 cortical cells were dissociated and transfected as described above. One day posttransfection, cultures were transferred to a Leica DMi8 microscope equipped with a live-cell incubation chamber maintained at 37°C and 5% CO_2_. Imaging was performed using a 20× air long–working-distance objective for a total duration of 80 hours, with images acquired every 15 min. Cells undergoing at least two successive divisions were tracked and quantified to determine cell cycle length and division mode.

### Cell cycle analysis

Cortices were dissected 1 day after IUE and dissociated into a single-cell suspension using a Pasteur pipette. Cells were stained with anti–mCD133-PE antibody as described above, followed by incubation with Hoechst 33342 Ready Flow Reagent (Invitrogen) at one drop per 10^6^ cells for 25 min at 37°C in the dark. Samples were analyzed on a FACSAria III Cell Sorter (BD Biosciences) using the FACSDiva software (version 6.1.3) as described above.

### CRISPR-KO

For the CRISPR-KO experiments of TGIF2a, the pSpCas9(BB)-2A-GFP (PX458, Addgene, #48138) plasmid was used to express the catalytically active form of *Streptococcus pyogenes* Cas9 (SpCas9) together with GFP under the control of the CAG promoter. gRNAs targeting *TGIF2a* were designed using Benchling (Benchling.com) and selected on the basis of high predicted on-target activity and minimal off-target risk using the Vienna BioCenter scoring system.

Two gRNAs targeting exon 2 of the *TGIF2a* isoform were selected (5′-CAGAGGCCTTACCCCCACGA-3′ and 5′-GTATTGAAGAGTCCACCCGT-3′) and cloned downstream of the human U6 promoter in a custom plasmid coexpressing the fluorescent protein mScarlet under the CAG promoter. A nontargeting gRNA (5′-GCTGCATGGGGCGCGAATCA-3′), predicted to have no genomic targets, served as the negative control and was cloned into the same plasmid backbone. For IUE, E13 mouse embryos were injected with a total of 1 μl of plasmid mix, containing PX458-Cas9-GFP (0.8 μg/μl) and 0.5 μg/μl of the mScarlet-U6-gRNA plasmid (either targeting or nontargeting).

### Western blot

For Western analysis, N2A cells (mouse neuroblastoma cells) were used. The cells were seeded in 10-cm dishes and transfected when they reached 65% confluency with 15 μg of DNA per dish (6 μg of overexpressing plasmids with either 25 nM siRNA pools or with 5 μg of mScarlet-U6-gRNA and 4 μg of PX458-Cas9-GFP). After 48 hours, cells were scraped and snap-frozen dry in dry ice and kept at –80°C. For protein isolation, cells were lysed in 1× radioimmunoprecipitation assay buffer containing cOmplete proteinase inhibitor. For Western blot analysis, 30 μg of protein was supplemented with 2× loading buffer, boiled at 95°C for 5 min, and spun down at full speed for 5 min at 4°C. Protein lysates were run on an 8% gel and blotted on nitrocellulose membranes. The antibodies used for blotting were anti-TGIF2 (1:500; Abcam, #ab222131), anti-vinculin (1:5000; Santa Cruz, #sc-73614), anti-rabbit HRP (1:5000; Life Technologies, 31458), and anti-mouse HRP (1:5000; Life Technologies, 31432).

### Sorting and bulk RNA-seq

To validate the centrality of TGIF2 in regulating CNPs, we sequenced NSCs after knocking out *Tgif2* with CRISPR in vivo. For IUE, E13 mouse embryos were injected with a total of 1 μl of plasmid mix, containing PX458-Cas9-GFP (0.8 μg/μl) and 0.5 μg/μl of the mScarlet-U6-gRNA plasmid (either targeting or nontargeting), and GFP+mScarlet+Prominin1+ NSCs were isolated by FACS sorting 48 hours after.

Brains were dissected in 1× HBSS (Gibco, catalog no. 14025) with 10 mM Hepes (Gibco, catalog no. 15630). The electroporated areas was visualized under an epifluorescent microscope and were dissected and centrifuged at 300*g* at 4°C for 5 min. Dissection buffer was aspirated, and tissue was enzymatically dissociated with 1 ml of 0.05% Trypsin/EDTA (Gibco, catalog no. 25300) for 15 min at 37°C. Digestion was inhibited by adding 2 ml of DMEM (Gibco, catalog no. 61965) with 10% FBS (PAN-Biotech, catalog no. P30-3302), and tissue was further mechanically dissociated with a fire-polished glass Pasteur pipette coated with DMEM + 10% FBS to obtain a single-cell suspension. The suspension was centrifuged at 300*g* at 4°C for 5 min, the supernatant was aspirated, and the cells were resuspended in 1× Staining Solution [1× HBSS, 1% glucose, 1 M Hepes, 1% FBS, 0.1% (w/v) NaN_3_, 1 mM EDTA, and DMEM-F12]. The cell suspension was stained with the preabsorbed antibody mCD133-allophycocyanin (APC) at 1:500 dilution (Anti-Mouse-CD133-APC, eBioscience/Invitrogen, catalog no. 12-1331-81). A corresponding isotype control antibody (Mouse IgM-APC, Miltenyi Biotec, catalog no. 130-093-176) was added to an isotype control sample in the same dilution. Cells were incubated at 4°C in the dark for 25 min, and then DAPI [1:1000 dilution of stock (1 mg/ml); Sigma-Aldrich, catalog no. D9542] was added, followed by another 5 min of incubation. To wash the cells, the suspension was filled up to 5 ml with PBS (Gibco, catalog no. 14190) and centrifuged at 300*g* at 4°C for 5 min. Cells were resuspended in PBS and filtered through a cell strainer (pluriStrainer Mini 40 μm, pluriSelect, catalog no. 43-10040-60) into suitable sample tubes (Falcon Round Bottom Polypropylene Test Tubes with Cap, Falcon, catalog no. 352063).

Cells were sorted on a FACSAria III Cell Sorter (BD Biosciences) with the FACSDiva software (version 6.1.3, BD Biosciences). To separate the populations, the first gate was set to separate small debris (low FSC) and dead or damaged cells, which were DAPI+ (high 450/40 signal). The second gate was set to remove doublets or cell aggregates by FSC-area/FSC-width. The third gate separated DAPI+ from DAPI– alive cells and the fourth gate the stained populations by the laser lines 660/20 for APC, with the gate set so that maximum of 0.2% of the parent population in the isotype control was detected as positive. Sorted cells were collected in RNA extraction buffer.

For the RNA-seq libraries, total RNA extraction was performed with the PicoPure RNA Isolation Kit (Applied Biosystems, catalog no. KIT0204) according to the manufacturer’s protocol. Genomic DNA was removed with the Zymo RNA Clean and Concentrator kit (R1013). RNA concentration and quality were evaluated on the Bioanalyzer (Model 2100, Agilent) using the RNA 6000 Pico Kit (Agilent, catalog no. 5067-1513) according to the manufacturer’s protocol. Samples with an RIN < 6.0 were excluded from library preparation. First-strand cDNA was prepared from 100 pg of RNA per sample with SMARTseq2 protocol, using SuperScript II Reverse Transcriptase (Invitrogen, #18064014) according to the manufacturer’s instructions. cDNA was amplified using a KAPA HiFi HotStart (Roche, 07958927001) and IS PCR primers with 18 amplification cycles.

The amplified cDNA was purified using AMPure XP magnetic beads (Beckmann Coulter, catalog no. QT650) and quality and quantity analyzed by Bioanalyzer (High Sensitivity DNA Kit, Agilent, catalog no. 5067-4626) and Qubit Assay (Qubit dsDNA HS Assay Kit and tubes, Invitrogen, catalog nos. Q32854/Q32856). Purified cDNA was fragmented by ultrasonic shearing on the Covaris AFA S220 system using corresponding tubes (microtube AFA Fiber Pre-Slit Snap-Cap 6x16mm, Covaris, catalog no. 520045), resulting in ~200- to 500-bp-long fragments that were purified by ethanol precipitation. Samples were evaluated again on the Bioanalyzer (HS DNA assay) before proceeding to the library preparation with the MicroPlex Library Preparation Kit v2 (Diagenode, catalog no. C05010014) according to the manufacturer’s instructions using 10 ng of cDNA per sample. Following the library amplification, cDNA concentration was verified by Qubit assay, and the libraries were purified over AMPure XP magnetic beads. Quality and quantity of these final libraries were evaluated by Bioanalyzer HS DNA assay, and samples were multiplexed at 5 nM each. Next-generation sequencing was performed on an Illumina HiSeq 1000 system with 60-bp paired-end deep sequencing.

The quality of sequencing data was analyzed with FastQC v0.12.1 (*59*). Reads were aligned to the mouse reference genome (mm39) using STAR v2.7.11b (*61*). Afterward, reads were deduplicated using picard MarkDuplicates, and gene expression was quantified with htseq-count v2.0.5 with parameters -m intersection-nonempty -f bam -r pos -s no --nonunique all -t transcript -i gene_id --additional-attr = gene_name. The subsequent analysis was performed in R version 4.4.1 (*63*). Genes with less than 10 counts across all samples were excluded. The expression data were normalized and transformed using the vst function of DESeq2 v1.44.0 (*64*) for plotting and outliers’ analysis. Samples from replicate 1 were removed from further analysis, as they clustered alone not with the rest of the samples (fig. S9K). DE analysis was performed using DESeq2 v1.44.0 (*64*). We tested for DE with DESeq2 using the Wald test and reported the genes with an FDR below 5% as significant. clusterProfiler was used for gene set enrichment analysis with all transcripts in the dataset as background. Data were plotted using the ggplot2 v3.5.1 package in R and the matplotlib v3.5.3 and seaborn v0.12.2 packages in Python.

### Statistical analysis

The statistical tests were performed using GraphPad Prism 9. If the data passed the Shapiro-Wilk normality test and *F* test (two conditions) or Barlett’s test (three or more conditions) for equal variance, then they were subject to either unpaired *t* tests when there were two conditions, or ordinary analysis of variance (ANOVA) with Tukey’s multiple-comparison test when there were three or more conditions. If the data passed the normality test but not equal variance, then they were subject to Welch *t* test when there are two conditions, or Brown-Forsythe and Welch ANOVA tests with Dunnett’s T3 multiple-comparison test when there were three or more conditions. If the data did not pass the normality test, then they were subject to Mann-Whitney test when there were two conditions, or Kruskal-Wallis ANOVA with Dunn’s multiple-comparison test when there were three or more conditions.
