# Supplementary material for: Transcriptional repression by TGIF2 coordinates neurogenic priming and neural stem cell maintenance
Source: Sci Adv. 2026 Jun 26;12(26):eaea9974. doi: 10.1126/sciadv.aea9974 (PMC13308598; doi:10.1126/sciadv.aea9974)
Supplement: Supplementary file 1 — Figs. S1 to S9 Legends for tables S1 to S10 [file sciadv.aea9974_sm.pdf]

Science Advances
AAAS

Supplementary Materials for

Transcriptional repression by TGIF2 coordinates neurogenic priming and neural stem cell maintenance

Yiling Li (李怡灵) *et al.*

Corresponding author: Magdalena Götz, magdalena.goetz@helmholtz-munich.de, magdalena.goetz@bmc.med.lmu.de

Sci. Adv. **12**, eaea9974 (2026)
DOI: 10.1126/sciadv.aea9974

The PDF file includes:

Figs. S1 to S9
Legends for tables S1 to S10

Other Supplementary Material for this manuscript includes the following:

Tables S1 to S10

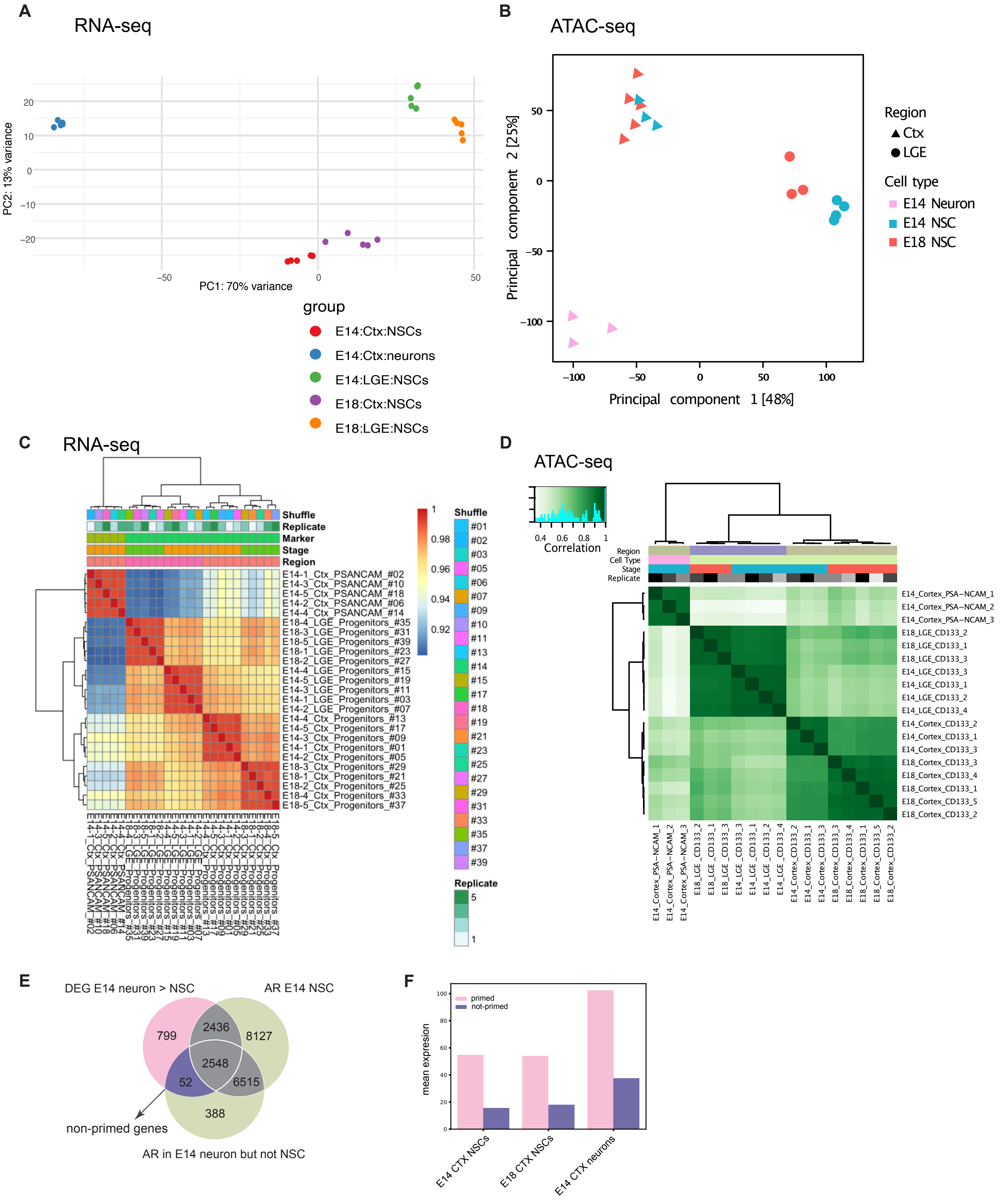
A
RNA-seq
PC2: 13% variance
PC1: 70% variance
20
10
0
−10
−20
−50
0
50
group
E14:Ctx:NSCs
E14:Ctx:neurons
E14:LGE:NSCs
E18:Ctx:NSCs
E18:LGE:NSCs
B
ATAC-seq
Principal component 2 [25%]
Principal component 1 [48%]
50
0
−50
−100
−100
−50
0
50
100
Region
Ctx
LGE
Cell type
E14 Neuron
E14 NSC
E18 NSC
C
RNA-seq
Shuffle
Replicate
Marker
Stage
Region
E14-1_Ctx_PSANCAM_#02
E14-3_Ctx_PSANCAM_#10
E14-5_Ctx_PSANCAM_#18
E14-2_Ctx_PSANCAM_#06
E14-4_Ctx_PSANCAM_#14
E18-4_LGE_Progenitors_#35
E18-3_LGE_Progenitors_#31
E18-5_LGE_Progenitors_#39
E18-1_LGE_Progenitors_#23
E18-2_LGE_Progenitors_#27
E14-4_LGE_Progenitors_#15
E14-5_LGE_Progenitors_#19
E14-3_LGE_Progenitors_#11
E14-1_LGE_Progenitors_#03
E14-2_LGE_Progenitors_#07
E14-4_Ctx_Progenitors_#13
E14-5_Ctx_Progenitors_#17
E14-3_Ctx_Progenitors_#09
E14-1_Ctx_Progenitors_#01
E14-2_Ctx_Progenitors_#05
E18-3_Ctx_Progenitors_#29
E18-1_Ctx_Progenitors_#21
E18-2_Ctx_Progenitors_#25
E18-4_Ctx_Progenitors_#33
E18-5_Ctx_Progenitors_#37
1
0.98
0.96
0.94
0.92
Shuffle
#01
#02
#03
#05
#06
#07
#09
#10
#11
#13
#14
#15
#17
#18
#19
#21
#23
#25
#27
#29
#31
#33
#35
#37
#39
Replicate
5
1
D
ATAC-seq
0.4
0.6
0.8
1
Correlation
Region
Cell Type
Stage
Replicate
E14_Cortex_PSA−NCAM_1
E14_Cortex_PSA−NCAM_2
E14_Cortex_PSA−NCAM_3
E18_LGE_CD133_2
E18_LGE_CD133_1
E18_LGE_CD133_3
E14_LGE_CD133_3
E14_LGE_CD133_1
E14_LGE_CD133_2
E14_LGE_CD133_4
E14_Cortex_CD133_2
E14_Cortex_CD133_1
E14_Cortex_CD133_3
E18_Cortex_CD133_3
E18_Cortex_CD133_4
E18_Cortex_CD133_1
E18_Cortex_CD133_5
E18_Cortex_CD133_2
E
DEG E14 neuron > NSC
AR E14 NSC
799
2436
8127
2548
52
6515
388
non-primed genes
AR in E14 neuron but not NSC
F
primed
not-primed
mean expresion
100
80
60
40
20
0
E14 CTX NSCs
E18 CTX NSCs
E14 CTX neurons

Fig. S1. RNA-seq and ATAC-seq data quality control

(A-B) Principal component analysis of the RNA-seq in (A) and ATAC-seq in (B) with region and stage marked with different colors and shapes. CTX: cortex; LGE: lateral ganglionic eminence; E: embryonic.
(C) Heatmap of RNA-seq samples clustered according to different parameters of the dataset.
(D) Heatmap of the ATAC-seq samples clustered based on chromatin openness and annotated based on different parameters of the dataset.
(E) Venn diagram showing the logic of identifying cortical non-primed genes.
(F) Histograms of mean expression between cortical neurogenic primed genes and non-primed genes in E14 and E18 cortical NSCs and E14 cortical neurons.

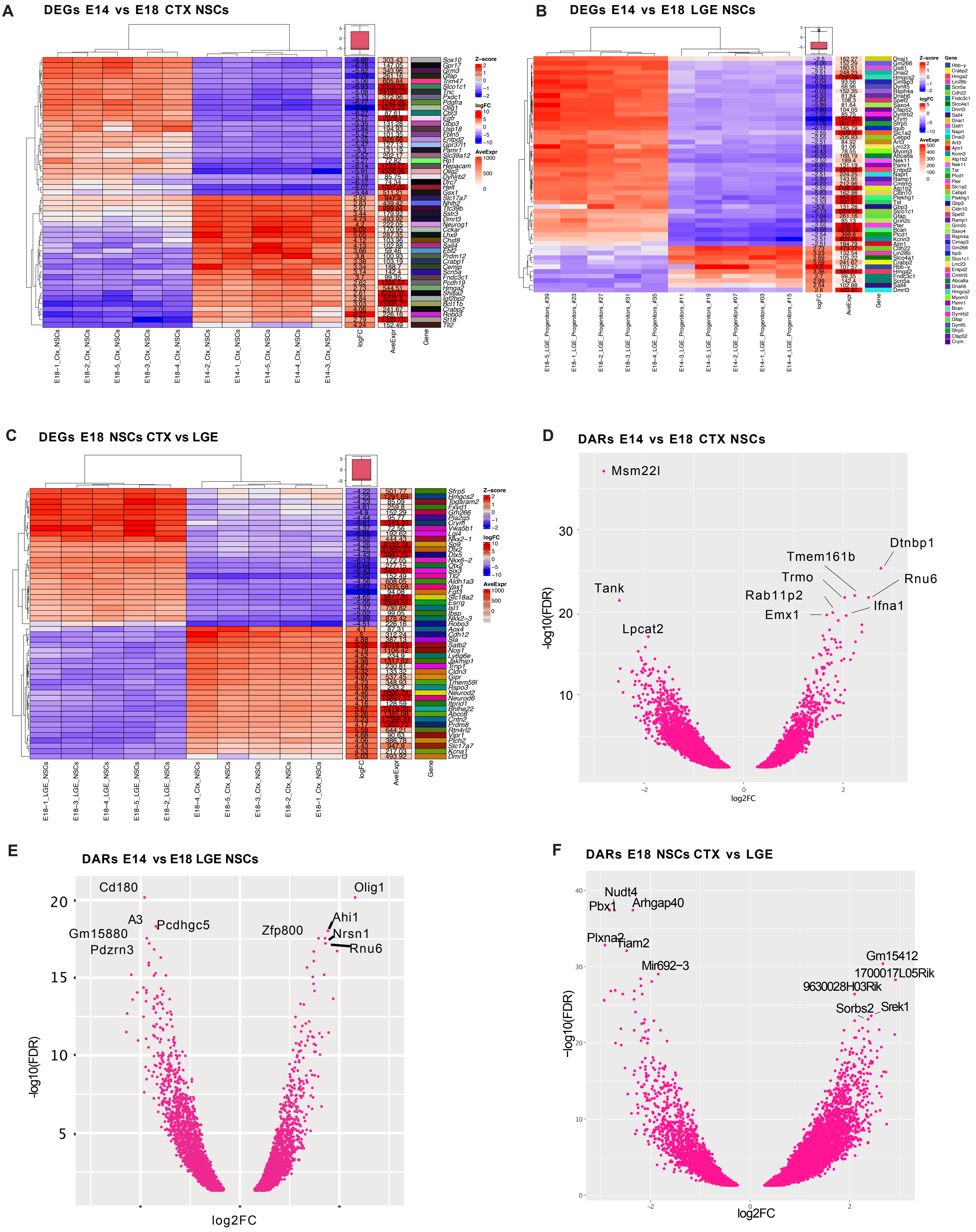
A
DEGs E14 vs E18 CTX NSCs
Z-score
logFC
AveExpr
Gene
E18-1_Ctx_NSCs
E18-2_Ctx_NSCs
E18-5_Ctx_NSCs
E18-3_Ctx_NSCs
E18-4_Ctx_NSCs
E14-2_Ctx_NSCs
E14-1_Ctx_NSCs
E14-5_Ctx_NSCs
E14-4_Ctx_NSCs
E14-3_Ctx_NSCs
logFC
AveExpr
Gene
B
DEGs E14 vs E18 LGE NSCs
Z-score
logFC
AveExpr
Gene
E18-5_LGE_Progenitors_#39
E18-1_LGE_Progenitors_#23
E18-2_LGE_Progenitors_#27
E18-3_LGE_Progenitors_#31
E18-4_LGE_Progenitors_#35
E14-3_LGE_Progenitors_#11
E14-5_LGE_Progenitors_#19
E14-2_LGE_Progenitors_#07
E14-1_LGE_Progenitors_#03
E14-4_LGE_Progenitors_#15
logFC
AveExpr
Gene
C
DEGs E18 NSCs CTX vs LGE
Z-score
logFC
AveExpr
E18-1_LGE_NSCs
E18-3_LGE_NSCs
E18-4_LGE_NSCs
E18-5_LGE_NSCs
E18-2_LGE_NSCs
E18-4_Ctx_NSCs
E18-5_Ctx_NSCs
E18-3_Ctx_NSCs
E18-2_Ctx_NSCs
E18-1_Ctx_NSCs
logFC
AveExpr
Gene
D
DARs E14 vs E18 CTX NSCs
Msm22l
Dtnbp1
Tmem161b
Trmo
Rnu6
Tank
Rab11p2
Ifna1
Emx1
Lpcat2
-log10(FDR)
30
20
10
-2
0
2
log2FC
E
DARs E14 vs E18 LGE NSCs
Cd180
Olig1
A3
Ahi1
Pcdhgc5
Gm15880
Zfp800
Nrsn1
Pdzrn3
Rnu6
20
15
10
5
-log10(FDR)
log2FC
F
DARs E18 NSCs CTX vs LGE
Nudt4
Pbx1
Arhgap40
Plxna2
Tiam2
Gm15412
Mir692-3
1700017L05Rik
9630028H03Rik
Sorbs2
Srek1
40
30
20
10
0
-log10(FDR)
-2
0
2
log2FC

Fig. S2. Differential gene expression and accessible regions.

(A) Heatmap of the top 25 differentially up- or down- regulated genes in the cortex at E14 versus E18. FC: fold change; AveExpr: average expression.

(B) Heatmap of the top 25 differentially up- or down- regulated genes in the LGE at E14 versus E18. FC: fold change; AveExpr: average expression.

(C) Heatmap of the top 25 differentially up- or down- regulated genes at E18 between cortex and LGE. FC: fold change; AveExpr: average expression.

(E) Volcano plot of significantly differentially accessible regions in E14 versus E18 NSCs in cortex (FDR < 0.05). Top 10 differential peaks are labeled by the nearest gene in their proximity. CTX: cortex; LGE: lateral ganglionic eminence; E: embryonic.

(E) Volcano plot of significantly differentially accessible regions in E14 versus E18 NSCs in LGE (FDR < 0.05). Top 10 differential peaks are labeled by the nearest gene in their proximity. CTX: cortex; LGE: lateral ganglionic eminence; E: embryonic.

(F) Volcano plot of significantly differentially accessible regions in cortex versus LGE at E18 (FDR < 0.05). Top 10 differential peaks are labeled by nearest gene in their proximity. Ctx: Cortex; LGE: Lateral Ganglionic Eminence; E: Embryonic.

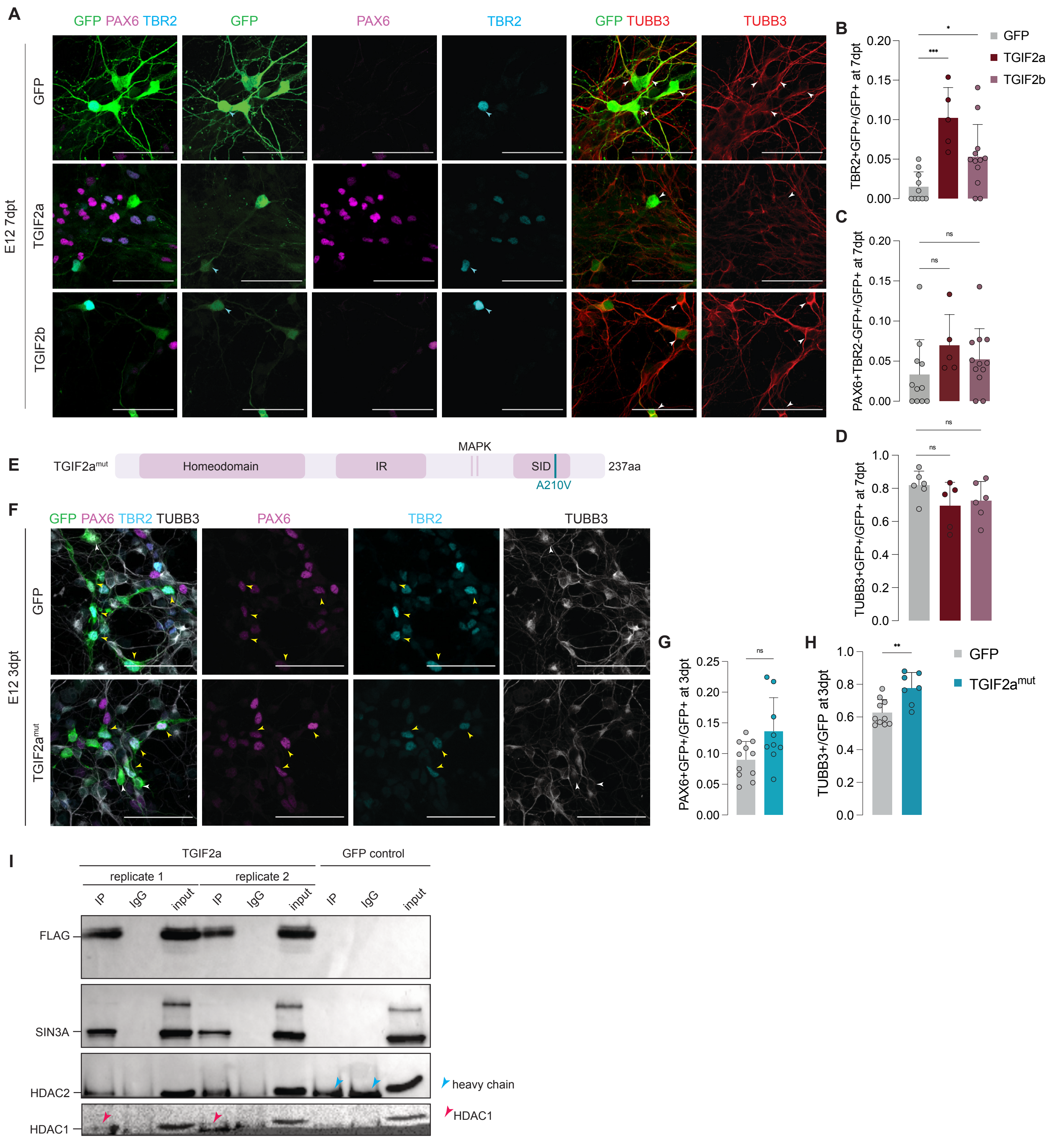
A
GFP PAX6 TBR2
GFP
PAX6
TBR2
GFP TUBB3
TUBB3
E12 7dpt
GFP
TGIF2a
TGIF2b
B
TBR2+GFP+/GFP+ at 7dpt
GFP
TGIF2a
TGIF2b
C
PAX6+TBR2-GFP+/GFP+ at 7dpt
D
TUBB3+GFP+/GFP+ at 7dpt
E
TGIF2amut
Homeodomain
IR
MAPK
SID
A210V
237aa
F
GFP PAX6 TBR2 TUBB3
PAX6
TBR2
TUBB3
E12 3dpt
GFP
TGIF2amut
G
PAX6+GFP+/GFP+ at 3dpt
H
TUBB3+/GFP at 3dpt
GFP
TGIF2amut
I
TGIF2a
GFP control
replicate 1
replicate 2
IP
IgG
input
FLAG
SIN3A
HDAC2
HDAC1
heavy chain

Fig. S3. TGIF2 expression during development and validations of TGIF2 KD.

(A) ISH data of mouse TGIF2 across different developmental timepoints, excerpts from Allen Brain Atlas.
(B) Tgif2 expression at E14 and E18, cortex and LGE from our RNA-seq. Significance was tested with two-way ANOVA with Benjamini, Krieger and Yekutieli correction.
(C) Bar plot showing Tgif2 isoform expression at E14 and E18 in cortical neural stem cells from our bulk RNA-seq. Tgif2a in dark red, Tgif2b in pink.
(D) Western blot analysis of siRNA pool knockdown efficiency against TGIF2. Isoform-specific overexpression plasmids were used to validate siRNA targeting, with protein levels normalized to vinculin as a loading control. N = 3 independent experiments. Ordinary one-way ANOVA with FDR correction for multiple comparisons test.
(E-F) Violin plots showing quantification of knockdown efficiency and titration with siRNA pool in final concentrations, together with 3xFlag-TGIF2a overexpression, measured by mean intensity in the channel of FLAG antibody In (c) and the channel of TGIF2 Abcam antibody in (D). N=8-21 cells measured with DAPI mask. Kruskal-Wallis ANOVA with Dunn's multiple comparisons test.
(G, H) Quantifications of TBR2+PAX6-GFP+/GFP+ cells at 3dpt, PAX6+TBR2-GFP+/GFP+ cells at 7dpt in LOF experiment, mean±SD. N = 3 pools of embryos. Two-tailed paired t-test. ns, not significant.
(I) Quantifications of TBR2+PAX6-GFP+/GFP+ cells at 3dpt in GOF experiment, mean±SD. N = 5-6 pools of embryos. Ordinary one-way ANOVA with Dunnett's multiple comparisons test. ns, not significant.

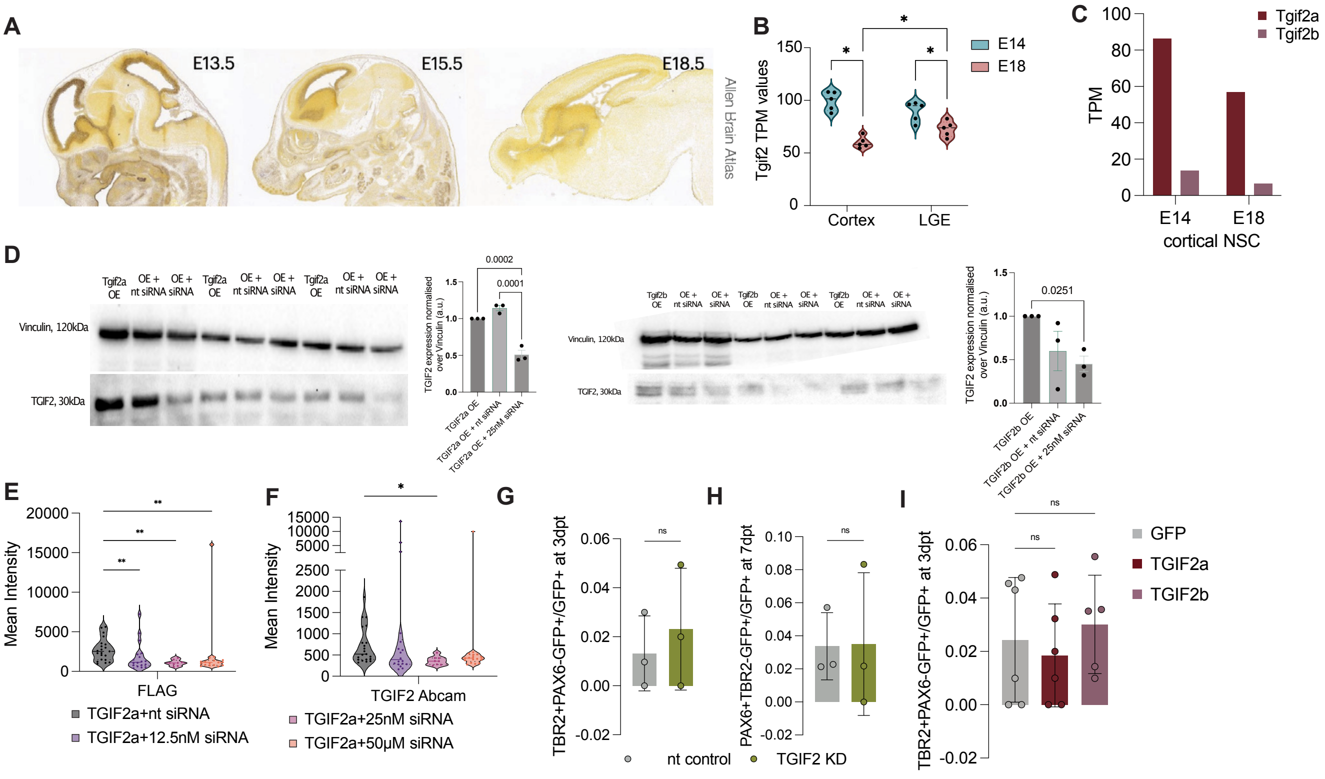

A
E13.5
E15.5
E18.5
Allen Brain Atlas
B
Tgif2 TPM values
Cortex
LGE
E14
E18
C
TPM
Tgif2a
Tgif2b
E14
E18
cortical NSC
D
Vinculin, 120kDa
TGIF2, 30kDa
TGIF2 expression normalised over Vinculin (a.u.)
0.0002
0.0001
0.0251
E
Mean Intensity
FLAG
TGIF2a+nt siRNA
TGIF2a+12.5nM siRNA
F
TGIF2 Abcam
TGIF2a+25nM siRNA
TGIF2a+50µM siRNA
G
TBR2+PAX6-GFP+/GFP+ at 3dpt
ns
H
PAX6+TBR2-GFP+/GFP+ at 7dpt
nt control
TGIF2 KD
I
TBR2+PAX6-GFP+/GFP+ at 3dpt
GFP
TGIF2a
TGIF2b

Fig. S4. TGIF2 effects on late-stage differentiation, domain perturbation analysis *in vitro*, and IP-WB validations of interactors

(A) Representative images showing E12 primary cortical cultures transfected with GFP or TGIF2 OE constructs at 7 days post transfection (dpt), co-stained with PAX6, TBR2, and TUBB3. Scale bar: 50µm. Cyan arrowheads indicate TBR2+PAX6-GFP+/GFP+ cells; white arrowheads indicate TUBB3+GFP+/GFP+ cells.
(B-D) Quantifications of TBR2+GFP+/GFP+ cells, PAX6+TBR2-GFP+/GFP+ cells, and TUBB3+GFP+/GFP+ cells at 7dpt, mean±SD. N = 5-12 pools of embryos. Ordinary one-way ANOVA with Dunnett's multiple comparisons test. * $p<0.05$, *** $p<0.001$. ns, not significant.
(E) Schematic drawing of TGIF2a^{mut} construct.
(F) Representative images showing E12 primary cortical cultures transfected with GFP or TGIF2a^{mut}, co-stained with PAX6, TBR2, and TUBB3. Yellow arrowheads indicate TBR2+PAX6-GFP+/GFP+ cells; white arrowheads indicate TUBB3+GFP+/GFP+ cells.
Scale bar: 50µm.
(G-H) Quantification of PAX6+/GFP+ and TUBB3+/GFP+ in E12 cortical cultures at 3dpt transfected with GFP or TGIF2a^{mut}, mean±SD. N= 7-11 pools of embryos. Mann-Whitney test.
(I) Western blots after co-immunoprecipitation of TGIF2a overexpression (OE) from 2 replicates and GFP OE in Neuro2A cells. Blue arrowheads indicate heavy chain contamination.

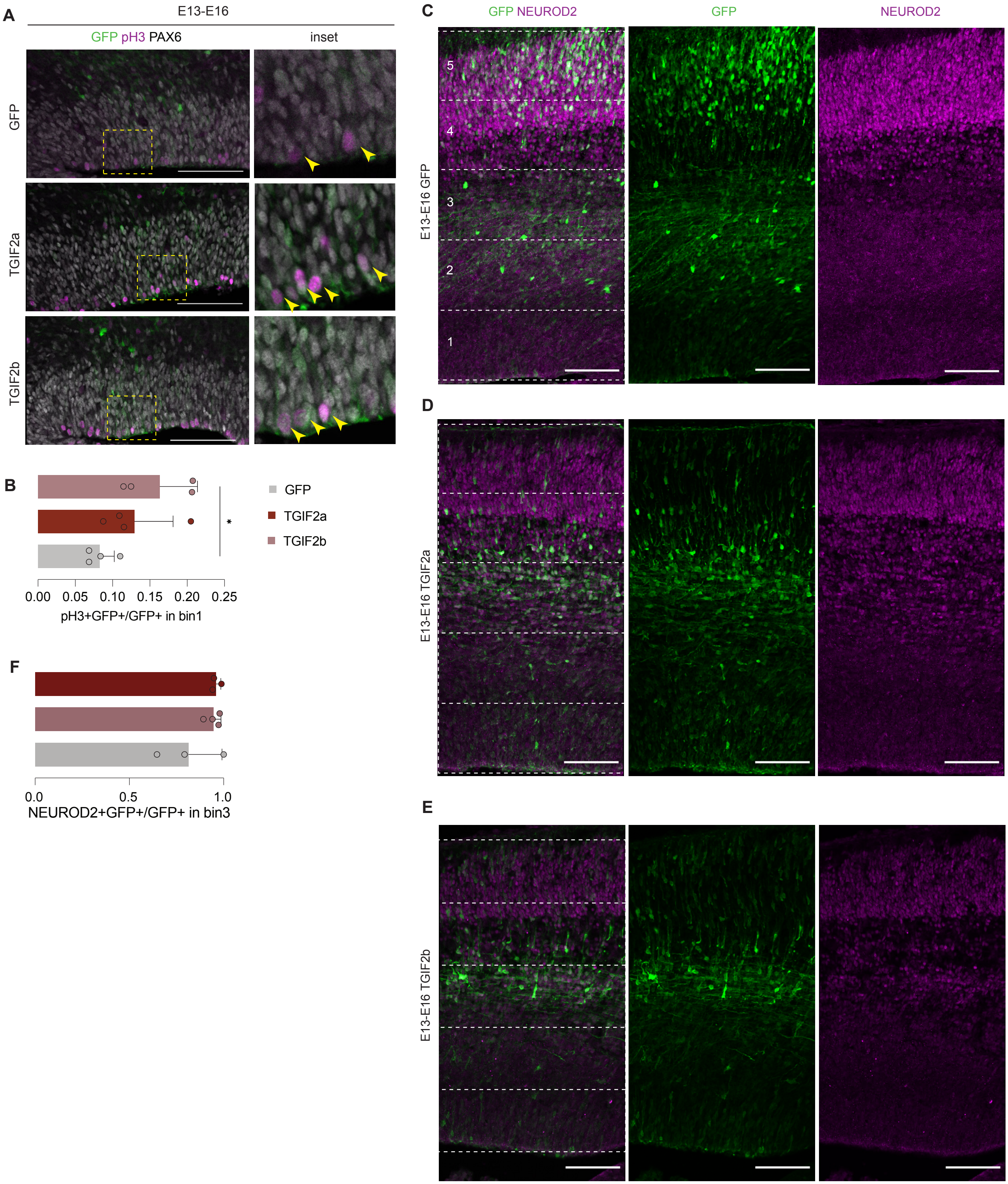

A
E13-E16
GFP pH3 PAX6
inset
GFP
TGIF2a
TGIF2b
B
GFP
TGIF2a
TGIF2b
*
0.00 0.05 0.10 0.15 0.20 0.25
pH3+GFP+/GFP+ in bin1
F
0.0 0.5 1.0
NEUROD2+GFP+/GFP+ in bin3
C
GFP NEUROD2
GFP
NEUROD2
5
4
3
2
1
E13-E16 GFP
D
E13-E16 TGIF2a
E
E13-E16 TGIF2b

Fig. S5. Immunostaining analysis after TGIF2 overexpression *in vivo*.

(A) Representative images and their insets of *in utero* electroporated cortices from different conditions (GFP+) immunostained with pH3 and PAX6. Arrowheads indicate pH3+GFP+/GFP+ cells. Scale bar: 100μm.

(B) Quantification of pH3+GFP+/GFP+ cells in bin 1, mean±SD. N=4 embryos from at least 2 mothers. Ordinary one-way ANOVA with Dunnett's multiple comparison's test.

(C-E) Representative images showing cortices 3 days post electroporation with different conditions in GFP, co-stained with NEUROD2. Dashed lines indicate the 5 equal bins. Scale bar: 100μm

(F) Quantification of NEUROD2+GFP+/GFP+ cells in bin 3, mean±SD. N=3-4 embryos from at least 2 mothers. Ordinary one-way ANOVA with Dunnett's multiple comparison's test, not significant.

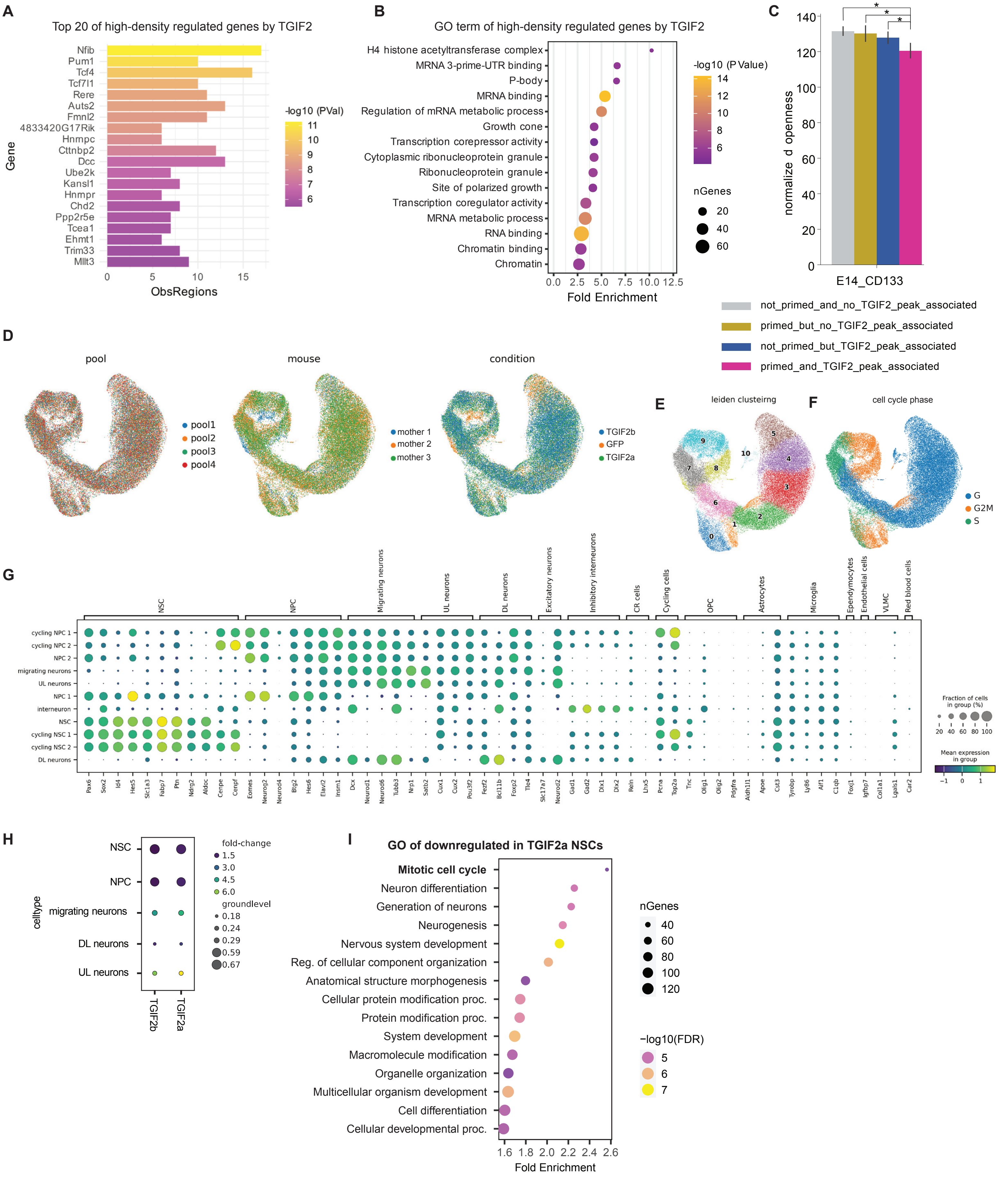
A
Top 20 of high-density regulated genes by TGIF2
Gene
Nfib
Pum1
Tcf4
Tcf7l1
Rere
Auts2
Fmnl2
4833420G17Rik
Hnmpc
Cttnbp2
Dcc
Ube2k
Kansl1
Hnmpr
Chd2
Ppp2r5e
Tcea1
Ehmt1
Trim33
Mllt3
ObsRegions
-log10 (PVal)
B
GO term of high-density regulated genes by TGIF2
H4 histone acetyltransferase complex
MRNA 3-prime-UTR binding
P-body
MRNA binding
Regulation of mRNA metabolic process
Growth cone
Transcription corepressor activity
Cytoplasmic ribonucleoprotein granule
Ribonucleoprotein granule
Site of polarized growth
Transcription coregulator activity
MRNA metabolic process
RNA binding
Chromatin binding
Chromatin
Fold Enrichment
-log10 (P Value)
nGenes
C
normalize d openness
E14_CD133
not_primed_and_no_TGIF2_peak_associated
primed_but_no_TGIF2_peak_associated
not_primed_but_TGIF2_peak_associated
primed_and_TGIF2_peak_associated
D
pool
pool1
pool2
pool3
pool4
mouse
mother 1
mother 2
mother 3
condition
TGIF2b
GFP
TGIF2a
E
leiden clusteirng
F
cell cycle phase
G
G2M
S
G
NSC
NPC
Migrating neurons
UL neurons
DL neurons
Excitatory neurons
Inhibitory interneurons
CR cells
Cycling cells
OPC
Astrocytes
Microglia
Ependymocytes
Endothelial cells
VLMC
Red blood cells
cycling NPC 1
cycling NPC 2
NPC 2
migrating neurons
UL neurons
NPC 1
interneuron
NSC
cycling NSC 1
cycling NSC 2
DL neurons
Pax6 Sox2 Id4 Hes5 Slc1a3 Fabp7 Ptn Ndrg2 Aldoc Cenpe Cenpf Eomes Neurog2 Neurod4 Btg2 Hes6 Elavl2 Insm1 Dcx Neurod1 Neurod6 Tubb3 Nrp1 Satb2 Cux1 Cux2 Pou3f2 Fezf2 Bcl11b Foxp2 Tle4 Slc17a7 Neurod2 Gad1 Gad2 Dlx1 Dlx2 Reln Lhx5 Pcna Top2a Tnc Olig1 Olig2 Pdgfra Aldh1l1 Apoe Cst3 Tyrobp Ly86 Aif1 C1qb Foxj1 Igfbp7 Col1a1 Lgals1 Car2
Fraction of cells in group (%)
Mean expression in group
H
celltype
NSC
NPC
migrating neurons
DL neurons
UL neurons
TGIF2b
TGIF2a
fold-change
groundlevel
I
GO of downregulated in TGIF2a NSCs
Mitotic cell cycle
Neuron differentiation
Generation of neurons
Neurogenesis
Nervous system development
Reg. of cellular component organization
Anatomical structure morphogenesis
Cellular protein modification proc.
Protein modification proc.
System development
Macromolecule modification
Organelle organization
Multicellular organism development
Cell differentiation
Cellular developmental proc.
Fold Enrichment
nGenes
−log10(FDR)

Fig. S6. Leiden clustering and marker gene expression in the scRNA-seq data
(A) Top 20 genes with high-density regulation by TGIF2a using GREAT.
(B) Top 15 enriched GO terms enriched in high-density regulated genes of TGIF2a.
(C) Chromatin accessibility comparison of peaks in proximity to primed genes and TGIF2 targets in E14 NSCs (CD133/Prominin1+), assessed by ATAC-seq mentioned in Fig. 1.
(D) UMAP projection of cells grouped by pool, mother, and condition.
(E) Leiden clustering with UMAP projection.
(F) Scatter plot of cell cycle phases from cell cycle marker gene expression.
(G) Dot plot of marker gene expression for each cell type.
(H) Dot plot showing Tgif2 expression fold change and ground level expression in overexpression conditions compared to the GFP control condition across different cell types.
(I) Top 15 GO terms enriched in downregulated genes in NSCs of TGIF2a condition.

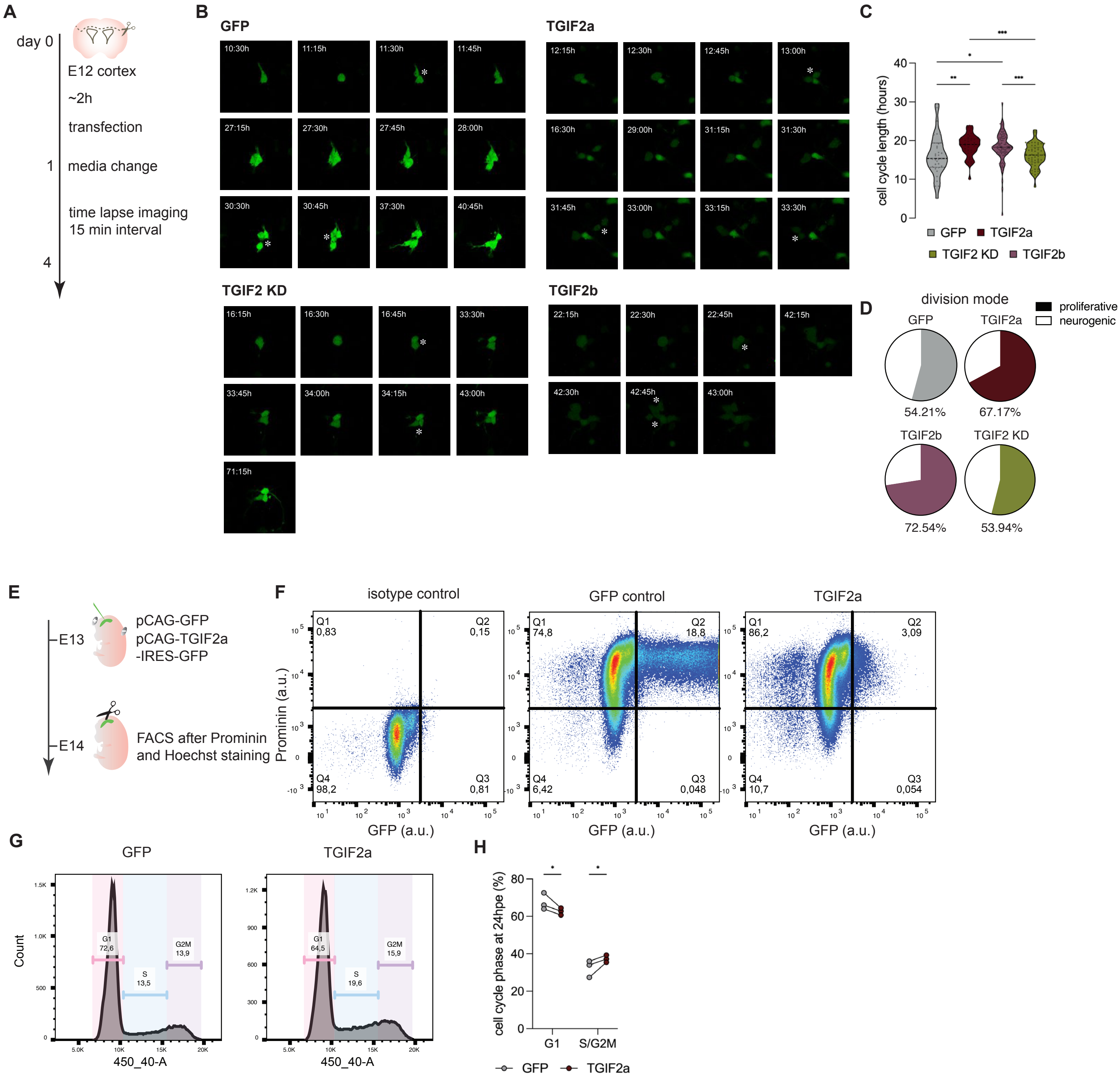
A
day 0
E12 cortex
~2h
transfection
1
media change
time lapse imaging
15 min interval
4
B
GFP
10:30h
11:15h
11:30h
11:45h
27:15h
27:30h
27:45h
28:00h
30:30h
30:45h
37:30h
40:45h
TGIF2a
12:15h
12:30h
12:45h
13:00h
16:30h
29:00h
31:15h
31:30h
31:45h
33:00h
33:15h
33:30h
TGIF2 KD
16:15h
16:30h
16:45h
33:30h
33:45h
34:00h
34:15h
43:00h
71:15h
TGIF2b
22:15h
22:30h
22:45h
42:15h
42:30h
42:45h
43:00h
C
cell cycle length (hours)
GFP
TGIF2a
TGIF2 KD
TGIF2b
D
division mode
proliferative
neurogenic
GFP
54.21%
TGIF2a
67.17%
TGIF2b
72.54%
TGIF2 KD
53.94%
E
E13
pCAG-GFP
pCAG-TGIF2a
-IRES-GFP
E14
FACS after Prominin
and Hoechst staining
F
isotype control
GFP control
TGIF2a
Prominin (a.u.)
Q1 0,83
Q2 0,15
Q4 98,2
Q3 0,81
Q1 74,8
Q2 18,8
Q4 6,42
Q3 0,048
Q1 86,2
Q2 3,09
Q4 10,7
Q3 0,054
GFP (a.u.)
G
GFP
TGIF2a
Count
G1 72,6
S 13,5
G2M 13,9
G1 64,5
S 19,6
G2M 15,9
450_40-A
H
cell cycle phase at 24hpe (%)
G1
S/G2M
GFP
TGIF2a

Fig. S7. Time-lapse live imaging and cell cycle analysis

(A) Experimental scheme of time-lapse live imaging using E12 cortical cultures.

(B) Examples pictures of time-lapse live imaging across different conditions. Star indicates cell division.

(C-D) Quantification of cell cycle length in violin plots and of division mode in pie chart. Proliferative: both of two daughter cells divide; neurogenic: one daughter divide, another daughter cell extends processes. N=3 pools of embryos from 3 mothers. Each dot in (c) represents one starter cell analyzed. Kruskal-Wallis test with Dunn's multiple comparisons test.

(E) Experimental scheme of cell cycle phase analysis using IUE and Hoechst staining.

(F) FACS gating strategy using un-electroporated cortices stained with isotype control of Prominin antibody.

(G) Examples of cell cycle phase analysis in GFP and TGIF2a condition based on DNA content from Hoechst staining.

(H) Quantification of cell cycle phase analysis. N=3 pools of embryos from 3 mothers. RM two-way ANOVA with two-stage step-up method of Benjamin, Krieger and Yekutieli.

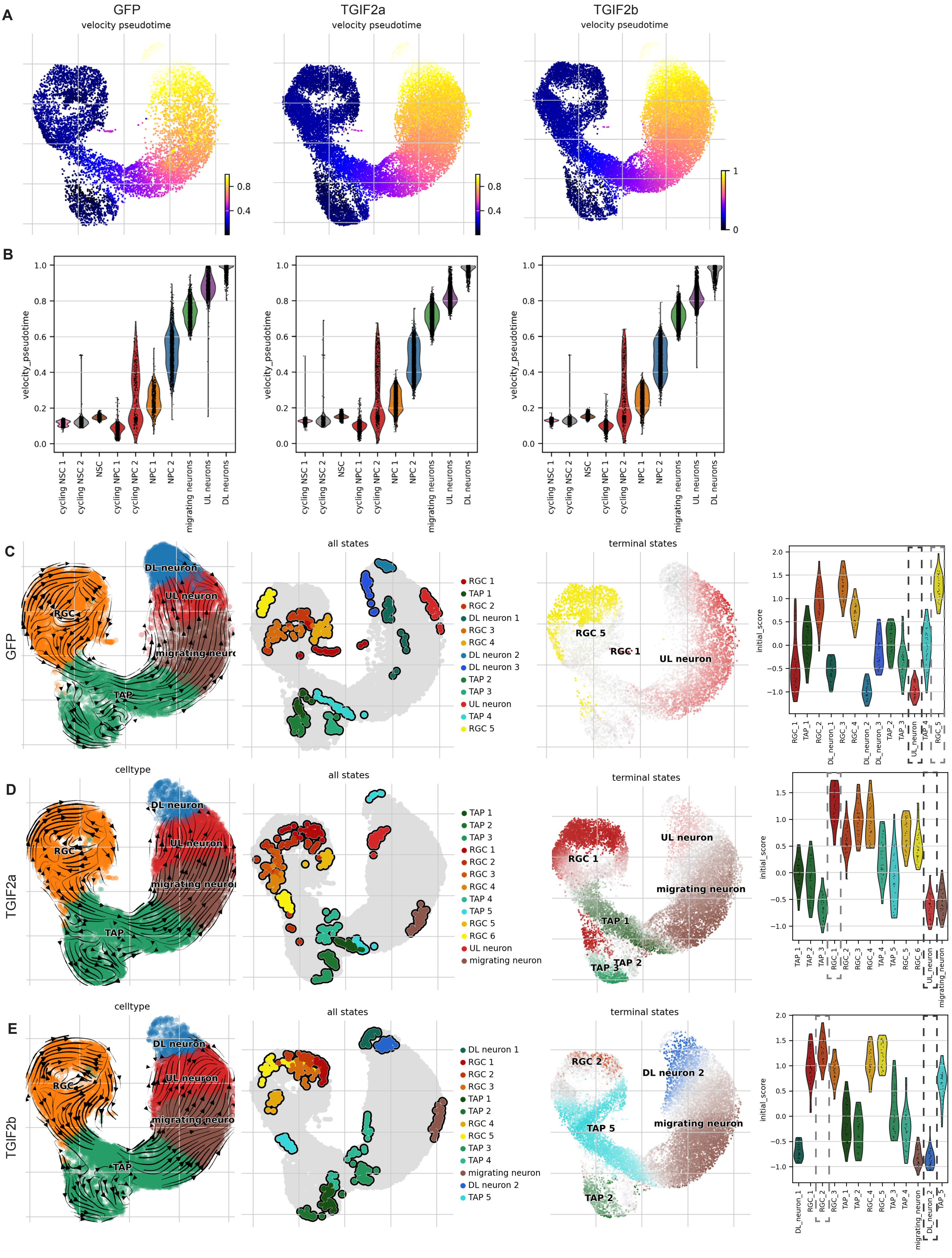
A
GFP
velocity pseudotime
TGIF2a
velocity pseudotime
TGIF2b
velocity pseudotime
B
velocity_pseudotime
cycling NSC 1
cycling NSC 2
NSC
cycling NPC 1
cycling NPC 2
NPC 1
NPC 2
migrating neurons
UL neurons
DL neurons
C
GFP
DL neuron
UL neuron
RGC
migrating neuro
TAP
all states
RGC 1
TAP 1
RGC 2
DL neuron 1
RGC 3
RGC 4
DL neuron 2
DL neuron 3
TAP 2
TAP 3
UL neuron
TAP 4
RGC 5
terminal states
RGC 5
RGC 1
UL neuron
initial_score
D
TGIF2a
celltype
DL neuron
RGC
UL neuron
migrating neuro
TAP
all states
TAP 1
TAP 2
TAP 3
RGC 1
RGC 2
RGC 3
RGC 4
TAP 4
TAP 5
RGC 5
RGC 6
UL neuron
migrating neuron
terminal states
RGC 1
UL neuron
migrating neuron
TAP 1
TAP 3
TAP 2
initial_score
E
TGIF2b
celltype
DL neuron
RGC
UL neuron
migrating neuro
TAP
all states
DL neuron 1
RGC 1
RGC 2
RGC 3
TAP 1
TAP 2
RGC 4
RGC 5
TAP 3
TAP 4
migrating neuron
DL neuron 2
TAP 5
terminal states
RGC 2
DL neuron 2
TAP 5
migrating neuron
TAP 2
initial_score

Fig. S8. Velocity pseudotime and CellRank procedures
(A) UMAP of velocity pseudotime across 3 conditions.
(B) Violin plots of velocity pseudotime across cell types in different conditions.
(C-E) UMAP representation of RNA velocity[24], macrostates, and terminal states predicted by CellRank[25,26]. Violin plots of initial score (Fabp7, Pax6, Sox2 expression) of macrostates predicted by CellRank. RGC: radial glial cells, or NSCS; TAP: transit amplifying progenitors, or NPCs.

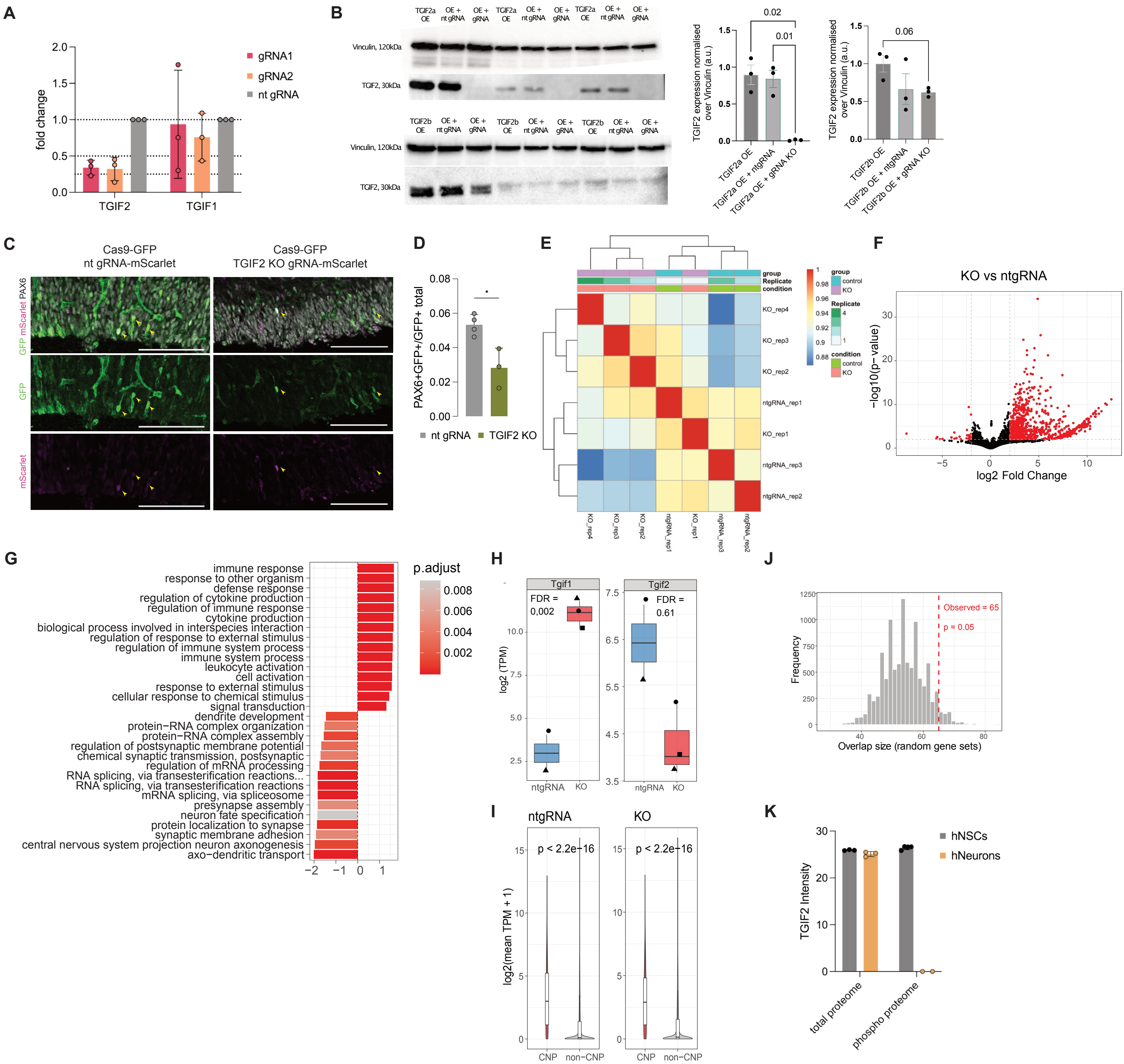

A
fold change
gRNA1
gRNA2
nt gRNA
TGIF2
TGIF1
B
Vinculin, 120kDa
TGIF2, 30kDa
TGIF2 expression normalised over Vinculin (a.u.)
TGIF2a OE
TGIF2a OE + ntgRNA
TGIF2a OE + gRNA KO
TGIF2b OE
TGIF2b OE + ntgRNA
TGIF2b OE + gRNA KO
C
Cas9-GFP nt gRNA-mScarlet
Cas9-GFP TGIF2 KO gRNA-mScarlet
GFP mScarlet PAX6
GFP
mScarlet
D
PAX6+GFP+/GFP+ total
nt gRNA
TGIF2 KO
E
F
KO vs ntgRNA
−log10(p−value)
log2 Fold Change
G
p.adjust
immune response
response to other organism
defense response
regulation of cytokine production
regulation of immune response
cytokine production
biological process involved in interspecies interaction
regulation of response to external stimulus
regulation of immune system process
immune system process
leukocyte activation
cell activation
response to external stimulus
cellular response to chemical stimulus
signal transduction
dendrite development
protein−RNA complex organization
protein−RNA complex assembly
regulation of postsynaptic membrane potential
chemical synaptic transmission, postsynaptic
regulation of mRNA processing
RNA splicing, via transesterification reactions...
RNA splicing, via transesterification reactions
mRNA splicing, via spliceosome
presynapse assembly
neuron fate specification
protein localization to synapse
synaptic membrane adhesion
central nervous system projection neuron axonogenesis
axo−dendritic transport
H
Tgif1
Tgif2
FDR = 0,002
FDR = 0.61
log2 (TPM)
ntgRNA
KO
I
ntgRNA
KO
p < 2.2e−16
log2(mean TPM + 1)
CNP
non−CNP
J
Observed = 65
p = 0.05
Frequency
Overlap size (random gene sets)
K
TGIF2 Intensity
hNSCs
hNeurons
total proteome
phospho proteome

Fig. S9. CRISPR-mediated TGIF2 knockout validation and TGIF2 protein phosphorylation status

(A) qRT-PCR analysis of N2A cells stably expressing Cas9 and transfected with two different TGIF2 knockout gRNAs targeting exon 2. Expression of Tgif2 and Tgif1 was measured, with Gapdh as a normalization control. Non-targeting (nt) gRNA served as a negative control.
(B) Western blot analysis of CRISPR KO efficiency against TGIF2. Isoform-specific overexpression plasmids were used to validate TGIF2 KO gRNA efficiency, with protein levels normalized to vinculin as a loading control. N = 3 independent experiments. Ordinary one-way ANOVA with FDR correction for multiple comparisons test.
(C) Representative pictures of IUE cortices electroporated with TGIF2 KO gRNA or nt guide in magenta, Cas9-GFP in green, and PAX6 in white. Arrowheads mark GFP+mScarlet+PAX6+ cells. Scale bar: 100µm.
(D) Quantification of PAX6+GFP+/GFP+ cells in bin 1, mean±SD. N=3-4 embryos from at least 2 mothers. Two-tailed unpaired t-test.
(E) Heatmap of RNA-seq samples clustered according to different parameters of the dataset.
(F) Volcano plot showing DEGs between KO and non-targeting (nt) control conditions.
(G) Gene set enrichment analysis of DEGs from (F).
(H) Expression levels of Tgif1 and Tgif2 by TPM in KO and nt control conditions.
(I) Mean expression levels of cortical neurogenic primed genes (CNP) and non-CNPs in KO and nt control conditions.
(J) Significance of overlap between DEGs from (F) and CNPs, determined by permutation testing (n=10,000 iterations).
(K) TGIF2 protein intensity in total and phospho-proteome data from human iPSC-derived NSCs and neurons[39].

Supplementary Tables.

Table S1. Cortical neurogenic primed genes and regulators

Table S2. DEG_Neurogenic NSCs regulators

Table S3. E14_vs_E18_in_Ctx_all_differential_peaks

Table S4. E14_vs_E18_in_LGE_all_differential_peaks

Table S5. Ctx_vs_LGE_in_E18_all_differential_peaks

Table S6. Neurogenic Fate Determinants

Table S7. List_of_shared_motifs

Table S8. Protein groups from IP-MS

Table S9. TGIF2 motif scanning and TGIF2 target genes

Table S10. DESeq_GSEA_KO vs control
